# Probabilistic Description of Streamflow and Active Length Regimes in Rivers

**DOI:** 10.1029/2021WR031344

**Published:** 2022-04-08

**Authors:** Nicola Durighetto, Veronica Mariotto, Francesca Zanetti, Kevin J. McGuire, Giuseppe Mendicino, Alfonso Senatore, Gianluca Botter

**Affiliations:** ^1^ Department of Civil, Environmental and Architectural Engineering University of Padua Padova Italy; ^2^ Department of Forest Resources & Environmental Conservation Virginia Tech Blacksburg VA USA; ^3^ Department of Environmental Engineering University of Calabria Rende Italy

**Keywords:** streamflow regimes, active length regimes, probabilistic description, temporary streams, stream length duration curve, SLDC

## Abstract

In spite of the prevalence of temporary rivers over a wide range of climatic conditions, they represent a relatively understudied fraction of the global river network. Here, we exploit a well‐established hydrological model and a derived distribution approach to develop a coupled probabilistic description for the dynamics of the catchment discharge and the corresponding active network length. Analytical expressions for the flow duration curve (FDC) and the stream length duration curve (SLDC) were derived and used to provide a consistent classification of streamflow and active length regimes in temporary rivers. Two distinct streamflow regimes (persistent and erratic) and three different types of active length regimes (ephemeral, perennial, and ephemeral de facto) were identified depending on the value of two dimensionless parameters. These key parameters, which are related to the underlying streamflow fluctuations and the sensitivity of active length to changes in the catchment discharge (here quantified by the scaling exponent b), originate seven different behavioral classes characterized by contrasting shapes of the underlying SLDCs and FDCs. The analytical model was tested using data gathered in three study catchments located in Italy and USA, with satisfactory model performances in most cases. Our analytical and empirical results show the existence of a structural relationship between streamflow and active length regimes, which is chiefly modulated by the scaling exponent *b*. The proposed framework represents a promising tool for the coupled analysis of discharge and river network length dynamics in temporary streams.

## Key Points

- We develop a stochastic model for the description of the statistics of discharge and active network length in rivers
- The model successfully described the observed statistics of discharge and active length in three case studies located in Italy and USA
- Two parameters with physical meaning were identified and used for developing a novel classification of streamflow and active length regimes

## 1 Introduction

The temporal evolution of the discharge observed at a station is modulated by a variety of complex eco‐hydrological processes that occur in the corresponding contributing catchment (Beven, 2012). In the light of the pronounced spatial heterogeneity of the hydrologic response of river basins (Betterle et al., 2019), classification tools have long been used in hydrology to synthesize the complex relationships that link the natural flow regime to landscape features and hydroclimatic variables. In particular, several quantitative frameworks have been proposed in the literature to classify the hydrologic dynamics observed at a given outlet using specific discharge metrics and/or physiographic/climatologic characteristics (Archfield et al., 2014; Berghuijs et al., 2014; Botter et al., 2013; Carrillo et al., 2011; Coopersmith et al., 2012; Kennard et al., 2010; Knoben et al., 2018; Kuentz et al., 2017; McManamay & Derolph, 2019; Poff et al., 1997; Sawicz et al., 2011; Wagener et al., 2007; Ye et al., 2012).

Most existing gauging stations, however, not only experience significant temporal variations in the observed discharge, but they also undergo dry periods during which the flow completely ceases (Datry et al., 2017; Messager et al., 2021; Zimmer et al., 2020). Temporary rivers (here broadly defined as streams characterized by a dry network portion sometime during the year) are in fact dominant features of arid, semi‐arid, and dry‐humid regions—but they are also ubiquitously observed in the headwaters of rivers characterized by wet or very wet climatic conditions (Durighetto et al., 2020; McDonough et al., 2011; Sauquet et al., 2020; Vander Vorste et al., 2020). Consequently, significant efforts have been made in recent years to extend tools and concepts originally conceived for the characterization of river flow regimes in perennial sections to temporary streams (Costigan et al., 2017). As a result, different classes of behavior have been defined based on quantitative metrics that summarize the hydrological and ecological conditions of a given reach. In particular, the attribution of a stream class is typically performed based on specific hydrologic indexes, including the number and duration of dry spells (Kaplan et al., 2019; Osterkamp & Hedman, 1982; Svec et al., 2005; Uys & O’Keeffe, 1997; Wohl, 2017), or using ad‐hoc ecomorphological indicators (Gallart et al., 2012; Gallo et al., 2020; Hansen, 2001; Leigh et al., 2016; Levick et al., 2018; Stromberg & Merritt, 2016; Stubbington et al., 2019). While existing classification approaches were mainly developed based on discharge records and in situ field inspections, hybrid frameworks that combine experimental observations and modeling tools can also be found in the literature (Williamson et al., 2015).

Existing classifications tools for temporary rivers share the adoption of a local perspective, according to which a stream type is associated to every single river segment (or reach) in a network, depending on the hydrologic and ecologic regime observed in that particular location. While extremely useful, reach‐wise classifications are difficult to extrapolate in space (Gordon & Goñi, 2003; Levick et al., 2018) as the resulting stream types might be highly heterogeneous along the stream network for example, owing to the internal geological complexity of a catchment (Durighetto & Botter, 2021). Moreover, local approaches do not take into account the status of the river network upstream of the focus stretch, which instead might be highly influential for a proper definition of the ecological and bio‐geochemical function of streams. The advantages implied by the adoption of a network‐scale perspective in the classification of temporary rivers have been already discussed in the literature (Gonzales‐Ferreras & Barquin, 2017), even though the practical efforts made by the scientific community in this direction are relatively limited.

The total active length of a channel network, *L*, and its temporal variability represent important spatially integrated characteristics of temporary streams. A proper characterization of the dynamics of *L* allows direct inference on the catchment‐scale effect of the expansion and contraction cycles experienced by the flowing network in response to unsteady hydro‐climatic conditions (Botter et al., 2021; Lapides et al., 2021). Besides, the active channel length and its temporal variations exert a fundamental control on a number of factors relevant for the temporal evolution of the stream water quality, including the distribution of catchment‐scale travel times (van Meerveld et al., 2019), the strength of hillslope‐channel connectivity (Soulsby et al., 2011) and the magnitude of important biogeochemical processes contributing to the metabolism of rivers (Bertuzzo et al., 2017; Botter, Basu, Zanardo, Rao, & Rinaldo, 2010; Kiel & Bayani Cardenas, 2014).

Even though the total active length of a temporary stream has been linked to the corresponding catchment discharge using empirical power‐law relationships (Godsey & Kirchner, 2014), a joint probabilistic description of the temporal variations in the hydrological response of a catchment and the corresponding changes in the active network length is lacking. Moreover, network‐scale classifications of temporary streams based on the total active length that is involved in the hydrologic response of a given catchment have never been developed.

In the light of these research gaps, in this article, we aim to provide a parsimonious probabilistic description of the coupled dynamics of the streamflow and the active length of a catchment. In doing that we specifically focus on the following research questions: (a) how is the temporal variability of the flowing length linked to the underlying streamflow regime? And (b) how can we provide a consistent network‐scale classification of temporary river networks combining information about flowing length and discharge regimes?

These research questions will be addressed by combining experimental observations gathered in three catchments located in different regions of the world with novel analytical derivations grounded on a well‐established streamflow probabilistic model.

## 2 Theory

### 2.1 Modeling Streamflow Statistics

River flow regimes are here quantified through the probability distribution function (pdf) of daily streamflows, using an analytical model which is based on a stochastic description of streamflow dynamics (Botter et al., 2007). These dynamics are assumed to result from the superposition of a sequence of flow pulses generated by precipitation. The model is well established in the literature and it has often been applied to estimate the pdf of daily flows based on limited information on climate and landscape (Basso et al., 2015; Botter et al., 2010, 2013; Doulatyari et al., 2015).

In this model, the rainfall hydrological forcing is described by a marked Poisson process with frequency *λ*_*p*_ [T^−1^] and exponentially distributed depths with average *α* [L]. Consequently, the sequence of events producing streamflow (i.e., effective rainfall) can also be approximated by a Poisson process, characterized by a frequency *λ* [T^−1^] (<*λ*_*p*_).

If the drainable catchment storage is assumed to behave as a linear reservoir with time constant *k* [T^−1^], the excess water deriving from the infiltrated rainfall, which represents the effective rainfall, is eliminated through the catchment hydrological response determining a sudden increase of the streamflow in correspondence of each event, followed by exponential recessions. Under the assumptions made, the magnitude of the jumps experienced by *q* are random and exponentially distributed with mean *αk*.

Provided that the system is linear, the overall streamflow is given by the sum of the contribution of the different effective pulses forcing the contributing catchment and the presence of overlapping pulses does not change the recession time constant, *k*. Hence, the stochastic dynamical equation for the specific discharge at a daily time scale reads (Botter et al., 2007):

(1) $$\frac{dq}{dt}=-kq\left(t\right)+\xi_{t},$$

where the temporal variations of specific discharge (per unit catchment area) $q\left(t\right)$ are equal to the sum of two terms: one expresses the exponential decay of the flow in between two events; the other, *ξ*_*t*_, describes the effect of a sequence of random streamflow increments due to effective rainfall events. Under these assumptions, the master equation associated with the stochastic process given in Equation 1 can be solved in steady‐state conditions. The corresponding analytical expression for the pdf of *q* is a Gamma‐distribution with scale parameter *λ*/*k* and rate parameter *αk* (Botter et al., 2007):

(2) $$p_{q}\left(q\right)=\frac{{\left(\alpha k\right)}^{-\frac{\lambda }{k}}}{\Gamma \left(\frac{\lambda }{k}\right)}\;q^{\frac{\lambda }{k}-1}\exp\left(-\frac{q}{\alpha k}\right),$$

where $\Gamma \left(\omega \right)$ is the complete Gamma‐function (Abramowitz & Stegun, 1964). The cumulative distribution function (cdf) of daily discharges can be calculated integrating the pdf of *q* given by Equation 2, as follows:

(3) $$P_{q}\left(q\right)=\frac{\gamma \left(\frac{\lambda }{k},\frac{q}{\alpha k}\right)}{\Gamma \left(\frac{\lambda }{k}\right)},$$

where *γ*(*ω*, *x*) is the lower incomplete Gamma‐function of argument *ω* with integration extreme equal to *x* (Abramowitz & Stegun, 1964).

Equation 3 represents the non‐exceedance probability of daily discharges during a reference period, expressed as a function of *α*, *λ*, and *k*. According to the above formulation, the mean discharge can be obtained through the following equation (Botter et al., 2007):

(4) 〈q〉=αλ.

Likewise, the coefficient of variation of daily discharges, *CV*_*q*_, can be analytically derived from Equation 2 as:

(5) $$CV_{q}=\sqrt{\frac{k}{\lambda }}.$$

This coefficient, which represents the ratio of the standard deviation to the mean, is a measure of dispersion of a probability distribution around the mean, and represents a quantitative metric to classify flow regimes as proposed by Botter et al. (2013). Equations 2 and 3 are best applied to individual seasons, as they rely on the assumption of stationarity.

### 2.2 Active Length Versus Streamflow Relationship

Streamflows and active network lengths of a river co‐evolve in response to the temporal variability of the climate forcings suitably modulated by landscape features. To evaluate the dynamicity of the river network, and quantify its tendency to extend or contract in response to rainfall events accounting for geologic and topographic characteristics of the site, a well‐established empirical relationship between the total active drainage length (*L*) and the corresponding specific discharge (*q*) was used. According to this formulation, the active stream length increases as a power law function of the catchment discharge as (Godsey & Kirchner, 2014; Jensen et al., 2017; Lapides et al., 2021; Senatore et al., 2021):

(6) $$L=\;a{\;q}^{b}.$$

In Equation 6, *a* [T^b^/L^b−1^] is a constant and *b* is the network scaling exponent, which typically ranges between 0.02 and 1. This analytical model for the description of the joint variations in active length and discharge has been already investigated and tested in several headwater networks in humid and semiarid climates (Blyth & Rodda, 1973; Day, 1978; Godsey & Kirchner, 2014; Gregory & Walling, 2010). Combining theoretical analysis and empirical data, the scaling exponent *b* was linked to physical properties of the catchment (Prancevic & Kirchner, 2019). In particular, *b* was found to be influenced by the exponents of other three geomorphological scaling relationships (local slope, valley transmissivity, and drainage area). These exponents depend on key characteristics of the study site such as the drainage density, the channel slope and the topographic curvature. Higher *b* values identify more dynamic stream networks.

In some cases, streamflow timeseries at the outlet of the catchment within which active lengths are evaluated might not be available, but discharge measurements could be available in a different control section of the same river. In such circumstances, a permanent streamflow could be observed even in cases where the river network within the focus catchment is completely dry. If the active network length is null when the discharge is strictly positive, Equation 6 needs to be replaced by the following formula (Senatore et al., 2021):

(7) $$L=\left\{\begin{array}{cc} {\left(q-q_{0}\right)}^{b}\; & \text{if}\;q>q_{0}\\ 0\; & \text{otherwise} \end{array}\right.$$

in which *q*_0_ represents a threshold specific discharge, for which *L* = 0.

### 2.3 The Stream Length Duration Curve (SLDC) as a Derived Distribution

The tool used in this study to characterize the degree of persistency of a river network is the SLDC. The SLDC, first introduced by Botter and Durighetto (2020), is defined as the inverse of the exceedance probability of the total active length of a stream and it can be seen as an analogous of the hydrological concept of flow duration curve (FDC). FDCs are largely used in the hydrological literature and in practical engineering to describe the temporal variability of river flows under particular site characteristics and hydrological features. They provide a useful basis to address important water resources problems such as water abstractions, environmental flows, hydropower, and irrigation (Ridolfi et al., 2020; Searcy, 1959; Vogel & Fennessey, 1994). Analogously, the SLDC enables the description of active stream dynamics providing a tool to compare stream extension and variability across different climatic areas.

The duration curve can be obtained graphically, by plotting the available active length data (*L*) on the vertical axis and the corresponding relative durations $\left(D\left(L\right)\right)$ on the horizontal axis. The duration of a given length expresses the time for which that flowing length is equaled or exceeded, and it is calculated as the complementary of the cumulative distribution function of *L* as follows:

(8) $$D\left(L\right)=1-P_{L}\left(L\right),$$

where *P*_*L*_(*L*) is the cumulative distribution function (i.e., the non exceedance probability) of the active length. The active length regime of a river can be defined based on the shape of the $D\left(L\right)$ curve, as discussed in the following sections.

To determine the SLDCs, the pdf of the active lengths, $p_{L}\left(L\right)$, or the related cumulative distribution function, $P_{L}\left(L\right)$, must be estimated. To this aim, a derived distribution approach that exploits the analytical relationship between *L* and *q* was used. Inverting Equation 6, it is possible to express *q* as a function of *L* as follows:

(9) $$q={\left(\frac{L}{a}\right)}^{\frac{1}{b}}.$$

Then, because *P*_*L*_(*L*) = *P*_*q*_(*q*(*L*)), the cumulative distribution of the active lengths can be obtained from Equation 3, as:

(10) $$P_{L}\left(L\right)=P_{q}\left(q\left(L\right)\right)=\frac{\gamma \left(\frac{\lambda }{k},\;\;{\left(\frac{L}{a}\right)}^{\frac{1}{b}}\frac{1}{\alpha k}\right)}{\Gamma \left(\frac{\lambda }{k}\right)}.$$

Equation 10 can be then differentiated in order to get the analytical expression for the pdf of *L*, which is a generalized Gamma‐distribution:

(11) $$p_{L}\left(L\right)=\frac{{\left(\alpha k\right)}^{-\frac{\lambda }{k}}}{ab\Gamma \left(\frac{\lambda }{k}\right)}{\left(\frac{L}{a}\right)}^{\frac{\lambda }{kb}-1}\exp\left(-{\left(\frac{L}{a}\right)}^{\frac{1}{b}}\frac{1}{\alpha k}\right).$$

The mean value for the active length is represented by the following formula:

(12) $$\langle L\rangle =\frac{a{\left(\alpha k\right)}^{b}\Gamma \left(\frac{\lambda }{k}+b\right)}{\Gamma \left(\frac{\lambda }{k}\right)},$$

and the modal value of the distribution reads:

(13) $$L_{0}=a{\left(\alpha k\right)}^{b}{\left(\frac{\lambda }{k}-b\right)}^{b}.$$

Hence, the ratio between the mode of the pdf of *L* (Equation 13) and the mean active length (Equation 12) is expressed as:

(14) $$\frac{L_{0}}{\langle L\rangle }=\frac{\Gamma \left(\frac{\lambda }{k}\right){\left(\frac{\lambda }{k}-b\right)}^{b}}{\Gamma \left(\frac{\lambda }{k}+b\right)}.$$

This ratio is useful as it is a measure of the asymmetry of the pdf of *L*. As an example, a ratio $\frac{L_{0}}{\langle L\rangle }\ll 1$ means that the preferential network length is much smaller than the mean. Under these circumstances, a major portion of the network activates only episodically, thereby impacting the mean length significantly without affecting the mode of the distribution.

Similarly, the coefficient of variation of the active length can be analytically derived as (Lapides et al., 2021):

(15) $$CV_{L}=\sqrt{\frac{\Gamma \left(\frac{\lambda }{k}\right)\Gamma \left(\frac{\lambda }{k}+2b\right)}{\Gamma {\left(\frac{\lambda }{k}+b\right)}^{2}}-1}.$$

This coefficient quantifies the extent of active length variations. When *λ*/(*kb*) ≪ 1, *p*_*L*_(*L*) defined in Equation 11 is monotonically decreasing, and pronounced network dynamics are observed (*CV*_*L*_ > 1). When *λ*/(*kb*) ≫ 1, *p*_*L*_(*L*) is bell‐shaped, and network dynamics are smoothed (*CV*_*L*_ < 1). The dependence of *CV*_*L*_ on *λ*/*k* and *b* will be further discussed in the following Section.

In some cases, a positive discharge may be associated with a null active length. These cases include, for example, the presence of a permanent spring at the outlet, or a situation in which streamflow and active length are measured at different locations. In these cases, analogous equations for the FDC and SLDC can be derived, by adding a positive, constant contribution to *q* in Equation 9 (see Appendix A).

### 2.4 Streamflow and Active Length Regimes

To identify the major drivers of discharge and active network length regimes, the analytical distributions of the catchment streamflow and active length have been normalized with the mean values of *q* and *L*. The pdf of the normalized discharge q* = *q*/〈*q*〉 can be written as:

(16) $${p^{*}}_{q}\left(q^{*}\right)=\frac{1}{\Gamma \left(\frac{\lambda }{k}\right)}{q^{*}}^{\frac{\lambda }{k}-1}\exp\left(-\frac{q^{*}\lambda }{k}\right).$$

which is only a function of *λ*/*k*. Likewise, the pdf of the normalized active length L* reads:

(17) $${p^{*}}_{L}\left(L^{*}\right)=\frac{1}{b}\frac{\Gamma {\left(\frac{\lambda }{k}+b\right)}^{\frac{\lambda }{kb}}}{\Gamma {\left(\frac{\lambda }{k}\right)}^{\frac{\lambda }{kb}+1}}{L^{*}}^{\frac{\lambda }{kb}-1}\exp\left(-{\left(L^{*}\frac{\Gamma \;\left(\frac{\lambda }{k}+b\right)}{\Gamma \left(\frac{\lambda }{k}\right)}\right)}^{\frac{1}{b}}\right),$$

where L* = *L*/〈*L*〉 is the ratio between active length values *L* and the mean active length 〈*L*〉, obtained through Equation 12.

Similarly, the cdfs of normalized *q* and *L* have can be represented by the following equations:

(18) $${P^{*}}_{q}\left(q^{*}\right)=\frac{\gamma \left(\frac{\lambda }{k},\frac{q^{*}\lambda }{k}\right)}{\Gamma \left(\frac{\lambda }{k}\right)},$$

and:

(19) $${P^{*}}_{L}\left(L^{*}\right)=\frac{\gamma \left(\frac{\lambda }{k},\;{\left(L^{*}\frac{\Gamma \;\left(\frac{\lambda }{k}+b\right)}{\Gamma \left(\frac{\lambda }{k}\right)}\right)}^{\frac{1}{b}}\right)}{\Gamma \left(\frac{\lambda }{k}\right)}.$$

Equations 16–19 clarify that the shape of the streamflow distribution depends on the ratio *λ*/*k* while the shape of active length distribution is controlled by two dimensionless parameters: *λ*/*k* and *b*.

**Figure 1:**
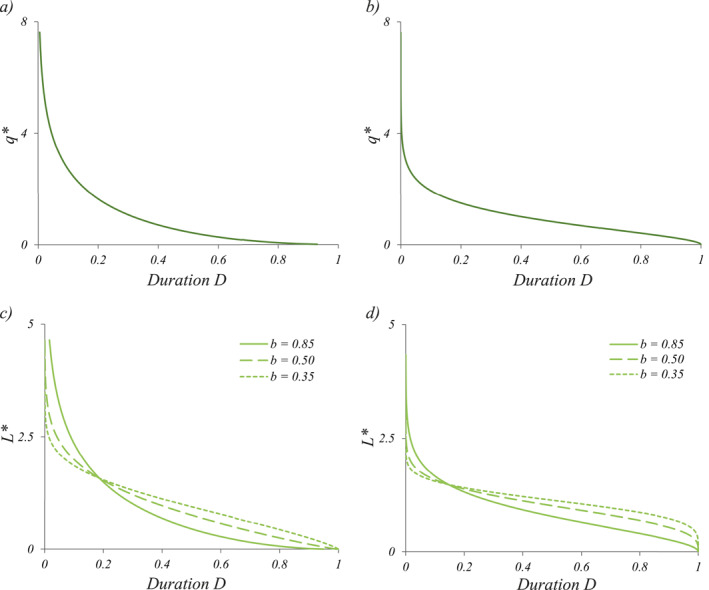
Theoretical examples of FDCs with normalized streamflow data (q* = *q*/〈*q*〉) and SLDCs with normalized active length data (L* = *L*/〈*L*〉) for erratic regime (panels (a and c) respectively, *λ*/*k* = 0.5) and for the persistent regime (panel (b and d) respectively, *λ*/*k* = 2). In panel (c), the values of *λ*/(*kb*) ranges from 0.59 (solid line) to 1.43 (dotted line), while in panel (d) they range from 2.35 (solid line) to 5.7 (dotted line). The term Duration refers to the fraction of time for which the corresponding q* and L* are equaled or exceeded.

A graphical representation of the FDC can be helpful in order to identify the different types of river flow regimes associated to different values of the dimensionless parameter *λ*/*k*. A convex duration curve that sharply decreases toward the *x*‐axis is obtained for *λ*/*k* < 1. This represents an erratic regime in which very low flows are frequent, but sporadic high flows are also possible (Figure 1a). The erratic regime is observed when the frequency of events producing streamflow (*λ*) is smaller than the recession time of the catchment (*k*). Under these circumstances, the river is able to dry considerably before the arrival of new pulses. Thus, streamflow strongly fluctuates in time and the preferential state of the system is lower than the mean. As per Equation 5, in the erratic flow regime the coefficient of variation of daily flows is larger than one (*CV*_*q*_ > 1).

Conversely, an S‐shaped FDC with an inflection point is obtained when *λ*/*k* > 1. This corresponds to a persistent regime with a limited streamflow variability and a continuous water supply to the stream from the contributing catchment (Figure 1b). This corresponds to cases in which the frequency of flow‐producing rainfall events (*λ*) is larger than the catchment recession rate (*k*). Under these conditions, the hydrograph is weakly variable around the mean discharge. Therefore, as per Equation 5, the coefficient of variation of daily flows is smaller than one (*CV*_*q*_ < 1) and the slopes of the FDC are gentler. FDCs typical of the intermediate regimes *λ*/*k* ≃ 1 exhibit a behavior which lies in between the two end members described above.

Differently from the flow regime, the shape of $P_{L}\left(L\right)$ and the corresponding active length regimes are driven by the interplay between *λ*/*k* and *b*. When *λ*/(*kb*) < 1, an ephemeral regime is obtained, and the river network is almost completely dry at some point during the reference period (e.g., one season, one year) but expands significantly in correspondence of a few rain events. The corresponding SLDC is a convex function of *D* which decreases sharply (Figure 1c) and is characterized by relatively high slopes (i.e., significant temporal variations of *L*). Note that if *λ*/*k* < 1, the SLDC can be concave, as the pdf of *q*, or it could exhibit an inflection point depending on the value of the parameter *b* (Figure 1c). For *λ*/*k* < 1 and *b* > 1, both the duration curves of *q* and *L* are convex functions of *D*. However, for a fixed value of *λ*/*k*, if the value of the scaling exponent *b* decreases the SLDC becomes flatter. Thus, if *λ*/*k* < 1 and *b* < 1, the SLDC and the FDC might be characterized by different shapes. When *λ*/(*kb*) > 1, in fact, the SLDC has an inflection point (e.g., Figure 1d), representing a perennial regime in which the shortest values of *L* are characterized by a duration of 1 (i.e., at least a portion of the stream network never dries out) and the temporal variations of *L* are relatively limited. However, in cases where the highest value of the probability density is observed for *L* → 0 (e.g., when *L*_0_/〈*L*〉 < 0.1), the active length regime can be defined as “ephemeral de facto” provided that most of the time the network is almost dry. Under these circumstances, the SLDC closely resembles a convex function of *D*, in which *D* ≃ 1 only for very low values of *L*. Hence, when *λ*/*k* > *b*, the actual behavior of the SLDC is determined by the ratio between the modal and the mean values of *L*, expressed by Equation 14. If this ratio is lower than, say, 0.1 the regime, though being perennial from a purely mathematical viewpoint, is ephemeral on practical grounds, provided that the preferential network configuration is much shorter than the mean network length. Numerical and analytical arguments suggest that, when *b* is lower than 1.0, the chance to observed an “ephemeral de facto” regime is quite limited. Thus, for *b* < 1 one can retain the distinction between ephemeral and perennial regimes previously introduced (if *λ*/*k* > *b* perennial; if *λ*/*k* < *b* ephemeral). For higher values of *b*, instead, the “ephemeral de facto” regime can be observed quite frequently, and needs to be explicitly considered. For practical reasons, Equation 14 can be approximated by a polynomial function. Thus, the criterion for identifying when the regime is ephemeral de facto can be written as:

(20) $$\frac{\lambda }{k}<0.4114\;b^{2}+0.7168\;b.$$

The above condition was obtained by imposing the condition *L*_0_/〈*L*〉 = 0.1 in Equation 14, and then numerically deriving the corresponding solution in the *λ*/*k* vs. *b* plane. The numerical solution was eventually fitted with a polynomial function of order two, which is shown on the r.h.s. of Equation 20. According to Equation 15, in the perennial regime *CV*_*L*_ < 1, whereas in both the ephemeral and ephemeral de facto regimes, *CV*_*L*_ > 1. Therefore, if *λ*/*k* < *b* an ephemeral regime for the active stream length is observed with *CV*_*L*_ > 1, while in cases where *λ*/*k* > *b* and Equation 20 is satisfied, the river shows an “ephemeral de facto” active length regime with *CV*_*L*_ > 1. Otherwise, if *λ*/*k* > *b* and the condition expressed by Equation 20 is not fulfilled, the river displays a perennial active length regime with *CV*_*L*_ < 1.

**Figure 2:**
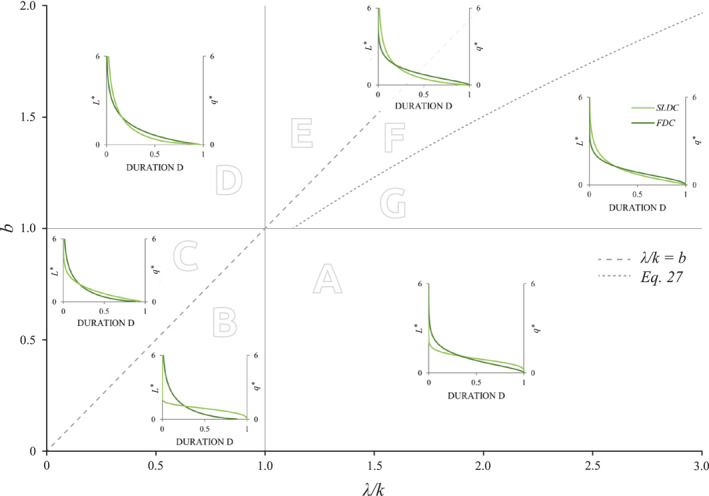
Theoretical classification streamflow and active length regimes. The classification is based on the shape flow duration curves (FDCs) and stream length duration curves (SLDCs), which is in turn controlled by the dimensionless parameters *b* and *λ*/*k*. The insets refer to the dimensionless discharge (q* = *q*/〈*q*〉) and active length (L* = *L*/〈*L*〉).

The analytical expressions derived above clarify how two dimensionless parameters, *λ*/*k* and *b*, determines different combinations of active length and discharge regimes in temporary rivers. The dependence of these regimes on the driving parameters, can be graphically represented in a *b* vs. *λ*/*k* plot (Figure 2).

As per the streamflow regimes, the plane can be subdivided into two regions: (a) when *λ*/*k* > 1, a persistent flow regime with an S‐shaped FDC is observed; (b) when *λ*/*k* < 1, instead, the flow regime is erratic, with a convex FDC. As per the active length regime, instead, three different domains can be identified in the *λ*/*k* vs. *b* plane. When *λ*/*k* < *b* the SLDC is steep and approaches the *x*‐axis for durations lower than 1, representing an ephemeral active length regime with relatively high probabilities associated with a river network that completely dries out. Conversely, if *λ*/*k* > 0.4114 *b* ^2^ + 0.7168 *b*, the SLDC results in a flat curve that covers the full range of durations. The S‐shaped SLDC indicates a perennial active length regime with a river network that never dries out. If *b* ≤ *λ*/*k* ≤ 0.4114 *b* ^2^ + 0.7168 *b*, the catchment experiences an ephemeral de facto active length regime, with a SLDC that intersects the *x*‐axis for *D* ≃ 1. In addition, if the value of *b* is lower (larger) than 1, the SLDC intersects the corresponding FDC due to its flatter (steeper) shape. Instead, if *b* is close to 1 the SLDC results similar to the corresponding FDC. Combining these conditions, we can thus identify seven macro‐regions or “classes”, which are characterized by different combinations of streamflow and active length regimes, as shown in Figure 2 and detailed in the following:

1. class A (*λ*/*k* > 1 and *b* < 1): in this class the flow regime is persistent and *CV*_*q*_ < 1. The active stream length regime is perennial, with *CV*_*L*_ < *CV*_*q*_ < 1. This corresponds to cases in which the variability of discharges is limited and so are the temporal changes in the active length. The temporal variability in the active length is very small and the river network includes a significant perennial portion that never dries out
2. class B (*λ*/*k* > *b* and *b* < 1): in this class the flow regime is erratic and *CV*_*q*_ > 1 but the active stream length regime is perennial, with *CV*_*L*_ < 1. This corresponds to cases in which the pronounced variability of the discharge is not mirrored by the timeseries of *L*, which exhibit limited temporal fluctuations. Accordingly, the river network has a perennial portion that never dries out
3. class C (*λ*/*k* < *b* and *b* < 1): in this class the flow regime is erratic and *CV*_*q*_ > 1. The active stream length regime is ephemeral, with *CV*_*Q*_ > *CV*_*L*_ > 1. This corresponds to cases in which the high variability of discharges is smoothed by the *L* time‐series. Nevertheless, the variability in the active length is pronounced and the river network tends to dry out for a non‐negligible portion of time, in correspondence of the lowest values of *q*
4. class D (*λ*/*k* < 1 and *b* > 1): in this class the flow regime is erratic and *CV*_*q*_ > 1. The active stream length regime is ephemeral, with *CV*_*L*_ > *CV*_*Q*_ > 1. This corresponds to cases in which the high variability of discharges is further enhanced by the active length dynamics. Thus, the variability in the active length is pronounced and the river network tends to dry out for a significant amount of time during the frequent droughts
5. class E (*λ*/*k* < *b* and *b* > 1): in this class the flow regime is persistent and *CV*_*q*_ < 1. The active stream length regime is ephemeral, with *CV*_*L*_ > 1. This corresponds to cases in which the variability of discharges is limited but significant temporal changes in the active length are observed. The pronounced variability in the active length through time is also reflected by the complete dry‐down of the river network. In fact, *L* = 0 for a non‐negligible portion of time, in correspondence of the lowest values of *q*
6. class F (*b* < *λ*/*k* < 0.4114 *b* ^2^ + 0.7168 *b* and *b* > 1): in this class the flow regime is persistent and *CV*_*q*_ < 1. The active stream length regime, instead, is ephemeral de facto, with *CV*_*L*_ > 1. This corresponds to cases in which the variability of discharges is limited but the corresponding temporal changes of the active length are comparatively more pronounced. The variability in the active length is moderate and the river network tends to dry out in correspondence of the lowest values of *q*. Mathematically, the most likely network configuration corresponds to a short network with a small but positive length. On practical grounds this regime is very similar to that identified as “class E”
7. class G (*λ*/*k* > 0.4114 *b* ^2^ + 0.7168 *b* and *b* > 1): in this class the flow regime is persistent and *CV*_*q*_ < 1. The active stream length regime is perennial, with *CV*_*q*_ < *CV*_*L*_ < 1. Although active length variations are more pronounced than the corresponding temporal changes of *q*, the active length variability is not huge. In fact, the river network includes some perennial reaches that never dry out

In some cases, differences in the shape of FDCs or SLDCs belonging to adjacent classes might be difficult to detect, owing to similarities in the steepness of the duration curves and the position of the inflection point (e.g., the FDC of classes A and G, or the SLDC of classes A and B in Figure 2). In any case, the proposed classification can be uniquely determined based on the underlying model parameters (*λ*, *k*, and *b*) and the implied values of the coefficients of variation of *q* and *L*, as described above.

## 3 Case Studies and Model Application

### 3.1 Case Studies

In this article, three rivers located in different regions of the world have been analyzed and used to describe the classification scheme presented above for streamflow and active length regimes. Two are Italian sites (Valfredda in the north and Turbolo in the south of Italy), while the third belongs to the southeastern USA (Poverty Creek).

In the following, a concise description of the test catchments is presented. The description includes also the origin of the data, the period of the analysis, and any detail necessary to replicate this study (See Table 1 for a summary).

**Table 1:** Summary of the Main Characteristics of the Study Sites

	Valfredda	Poverty Creek	Turbolo
Area of the catchment [km^2^]	2.60	0.33	0.67
Rain station	Valfredda	KVABLACK18	Fitterizzi
Rain station within the catchment	Y	N	N
Discharge data available at the outlet	Y	Y	N
Maximum active length [km]	2.00	1.80	2.70
Active network mapping	Sensors	Sensors	Field surveys
Period	09/2019–10/2019	04/2017–02/2018	04/2019–01/2020
Subperiods	1	4	4

**Figure 3:**
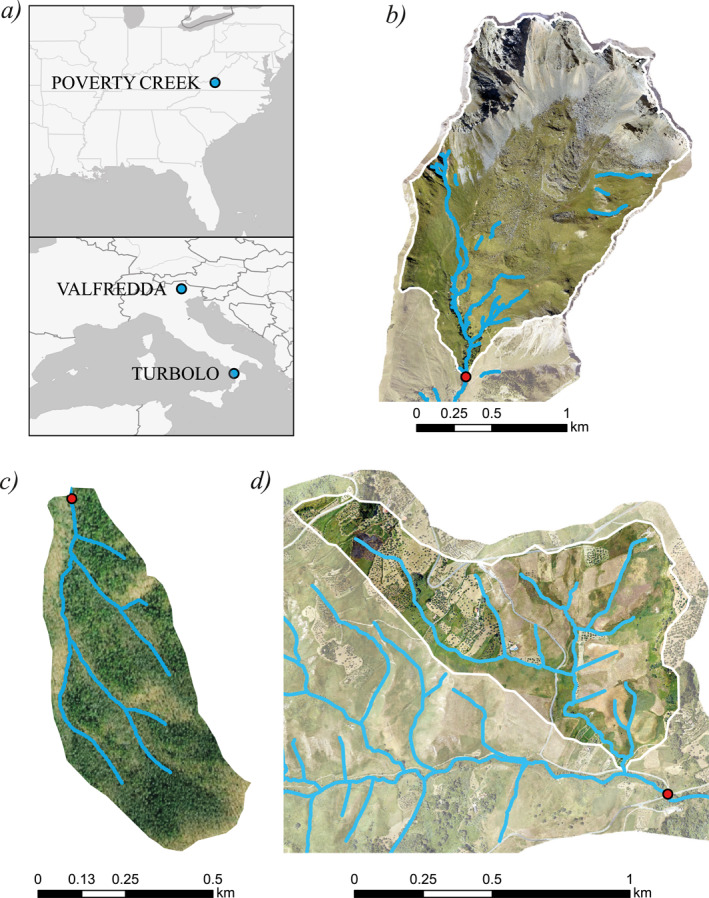
Location of the study catchments in Europe and eastern USA (a). Morphology of the Rio Valfredda (a), Poverty Creek (b) and Turbolo Creek (c). Blue lines show the potential stream network; the location where streamflow was measured is marked with red dots.

The Rio Valfredda is an alpine creek located in the province of Belluno, in the northern Italy, on the border between two regions: Veneto and Trentino Alto Adige (Figure 3a). The study catchment, which belongs to Piave river basin, has an overall area of 2.6 km^2^ and its elevation ranges from 1,900 m a.s.l. to 3,000 m a.s.l. This layout, which involves areas placed at very different heights, provides a very complex and diversified morphology. In fact, the soil composition and the vegetation features change along the altimetric gradient of the catchment (Figure 3a). The Valfredda catchment has been extensively studied in previous work (Botter & Durighetto, 2020; Durighetto & Botter, 2021; Durighetto et al., 2020; Zanetti et al., 2021). The Valfredda river has an alpine climate, characterized by cold snowy winters and wet summers. The lowest flows are observed in the winter, while the higher discharges occur during spring and summer caused by both precipitation and snow melting (Durighetto et al., 2020). Climate data were collected by a meteorological station located near the centroid of the contributing catchment. Streamflow data have been taken in a controlled cross‐section at the outlet of the study catchment while wet length data have been calculated exploiting a network of 31 water presence sensors which were placed along the potential stream network with an average spacing of about 40 m. These sensors collected data every 5 min about the presence/absence of flowing water at the location where each sensor was deployed. The data refer to the fall of 2019, and are discussed in detail in Zanetti et al. (2021).

The Poverty Creek is a river located in the Jefferson National Forest of Montgomery Country in Virginia, USA (Figure 3b). The catchment has an area of 0.33 km^2^ and its elevation ranges between 660 and 830 m a.s.l. The geology and the land cover are relatively homogeneous within the catchment. The climate of the site is characterized by cold winters with rare snowfall. Springs and summers are characterized by high temperatures, with a few intense rainfall events. In this type of climate, the annual maximum flow is usually observed during the winter and early spring (Jensen et al., 2017, 2019). The available rainfall and temperature values have been collected by a weather station (Weather Underground KVABLACK18) located on the Brush Mountain, at a distance of 3 km from the catchment centroid. Streamflow measurements were undertaken at the outlet of the contributing catchment from stage data through a rating curve built using the salt dilution method (Calkins & Dunne, 1970). Wet length data have been obtained using 51 flow intermittency sensors deployed with an average spacing of 40 m during several field surveys carried out from April 2017 till February 2018 (Jensen et al., 2019).

The third study site is a small headwater catchment which feeds the upper portion of the Turbolo creek, a tributary of the Crati River in southern Italy (Figure 3c). At the outlet of Fitterizzi, the Turbolo catchment has an overall area of 7 km^2^, with an elevation between 183 m a.s.l. and 1,005 m a.s.l. The lithology of the basin is composed mainly by fractured rocks which enhance the soil storage capacity and permeability. Such conditions allow relevant groundwater storage leading to almost perennial flow at the catchment outlet of Fitterizzi. The area is characterized by a Mediterranean climate with hot and dry summers and wet and mild winters, with a relatively high rainfall frequency. In this study, only the East subcatchment of the Turbolo river, evidenced in Figure 3d, was considered. This subcatchment has an area of 0.67 km^2^, and its lithology is mainly characterized by silty marly clays with poor erosion resistance and low permeability, and overlapping sandy‐conglomerate formations. Climate data were collected by a weather station, managed by ARPACal (Calabria Region Protection Agency). The weather station is 200 m far from to the catchment outlet, and therefore it is deemed to be highly representative of the weather conditions of the area. The active length data come from field surveys carried out between April 2019 and January 2020 (Senatore et al., 2021). The visual field surveys consisted in walking along the river network, collecting GPS coordinates of the points where surface flow started or stopped. As discharge data at the outlet of the East subcatchment were not available, streamflow data gathered through a water stage gauge installed at the outlet of Fitterizzi were used in this study.

The three case studies are characterized by diverse landscape, climatic and hydrological features (i.e., rainfall, geology, river network characteristics). For example, in the area of Valfredda creek, the temperature varied between −0.6°C and 24.7°C, while precipitation rates varied from 0 to 35.2 mm per day. In the Poverty Creek, mean temperatures varied from −4°C recorded in January to the highest 22°C recorded in July, while precipitation ranged between 0 and 66.3 mm per day. Regarding the Turbolo creek, the lowest observed value of temperature is −2.6°C in January while the highest one is 38.4°C observed during August, and precipitation rates peak at 81.8 mm per day. The observed inter‐catchment variability of land cover is also pronounced. Testing the model under a broad range of conditions in terms of climate, geology, and morphology allows the assessment of the reliability of the proposed model under diverse settings.

### 3.2 Model Application and Evaluation

The model application consisted of three different and sequential steps: (a) parameter estimation; (b) calculation of streamflow and active length statistics; (c) model evaluation.

As per the estimation of the streamflow model, the reference parameters *α*, *λ*, and *k* were estimated on a seasonal basis starting from the available data of daily rainfall [L] and daily specific discharge [L/T]. *k* was estimated using recession data, as detailed in Basso et al. (2015). For the identification of *λ*, two methods were used: (a) a mass balance between rainfall and streamflow: *λ* = 〈*q*〉/*α* (Equation 4); (b) a computation of the frequency of positive jumps observed in the streamflow series (Betterle et al., 2017; Ceola et al., 2010). The method (b) was used only in cases where several outliers in the observed streamflow data were detected (e.g., Poverty Creek during winter and spring season), which might impair the discharge sample mean.

The parameter *α* was calculated in two different ways: from rainfall data (method c) or using a statistical calibration (method d). Generally, the parameter *α* was calculated as the average of the daily precipitation depths observed during rainy days. In some cases, however, unrealistically high values of *α* were obtained with this method (c)—an instance which indicates some inconsistency between rainfall and discharge data. In these cases, adjusting the streamflow data was unfeasible because of the difficulties in characterizing systematic errors in discharge measurements. Consequently, the inconsistency was resolved by calibrating *α* and minimizing the model errors in reproducing the relevant quantiles of the distribution (method d).

The parameters *a* and *b* of Equation 6 instead were obtained by a linear regression in the log(*L*) − log(*q*) plane starting from available *L* and *q* data (see Supporting Information S1). The constant *a* was determined as the antilog of the intercept value, while the exponent *b* was calculated as the slope of the regression line. When Equation 7 was used, the same procedure described above was applied replacing *q* with (*q* − *q*_0_), where *q*_0_ corresponds to the maximum specific discharge for which *L* = 0. In all cases, the goodness of fit between the available data has been estimated in the linear regression through the *R* ^2^ coefficient.

The calculation of the underlying statistics of *L* and *q* was performed using the analytical equations detailed in Section 2, exploiting an ad‐hoc Matlab code. The pdfs and cdfs of *q* and *L* are first evaluated at the seasonal level, and then—when needed—they were aggregated at the annual timescale through a weighted average of the corresponding seasonal distributions (Supporting Information S1). The seasons have been defined on a calendar basis, taking into account the duration of the available timeseries, as detailed below. The subdivision into seasons using fixed calendar dates represents the standard for the application of the stochastic streamflow model used in this article (see e.g., Basso et al., 2015; Botter et al., 2013). In some cases, however, this standard subdivision poses concerns when applied to individual hydrologic years, during which the observed seasonality might differ from the long‐term behavior of the study site. In such circumstances (e.g., the winter season in the Poverty), additional subperiods within individual seasons might be needed to avoid poor performances of the model, which would be simply related to climatic patterns within individual seasons. In the light of the limited length of the data set available in the Valfredda catchment, we avoided any unnecessary subdivision into standard seasons in that case.

The robustness of the analytical model for the probabilistic description of *q* and *L* developed in this article was assessed by comparing the analytical pdfs (and cdfs) of *q* and *L* with the corresponding empirical distributions of observed discharges and active lengths. The empirical pdfs of *q* and *L* were created subdividing the range between zero and the maximum observed value of *q* and *L* into a number of equally spaced intervals, and then computing the frequency distribution of the observations across these intervals. The empirical cdfs, instead, have been calculated using a standard Weibull plotting position, after a suitable ranking of the data based on their magnitude.

Finally, the performance of the models was evaluated for each case study and each subperiod of analysis using the mean absolute error (MAE). The MAE is calculated as the mean of the absolute difference between the analytical and the observed quantiles of the cdfs of *q* and *L* evaluated at the 0.2, 0.4, 0.6, and 0.8 quantiles. The analytical quantiles were calculated using expressions for *P*_*q*_(*q*; Equation 3) and *P*_*L*_(*L*; Equation 10 or Equation A2). The MAE was also scaled to the mean value of the corresponding distribution (i.e., scaled mean absolute error, SMAE).

## 4 Results

Starting from the available hydroclimatic data, the parameters of the analytical model have been estimated for each case study, as shown in Table 2. These parameters enabled the calculation of the analytical distributions of *q* and *L*, which were then compared with the corresponding empirical counterparts, as discussed in the following.

**Table 2:** Summary of the Fitted Parameters for the Different Case Studies of This Article

Catchment	Period	*α* [cm]	*λ* [d^−1^]	*K* [d^−1^]	Fitting method	*q*_0_ [cm/d]	*a* [d^b^/cm^b−1^]	*b* [ − ]	*R* ^2^ [ − ]
Valfredda	Autumn	3.00	0.14	0.05	a–d	‐	0.40	0.17	0.52
Poverty Creek	Spring	9.00	0.32	0.14	a–d	‐	0.30	1.09	0.77
	Summer	0.72	0.23	0.19	a–c	‐			
	Autumn	0.92	0.25	0.20	a–c	‐			
	Winter 1	0.50	0.29	0.12	a–d	‐			
	Winter 2	6.00	0.34	0.14	a–d	‐			
Turbolo	Spring	0.52	0.20	0.07	a–c	‐	6.17	0.50	0.21
	Summer	0.91	0.04	0.06	a–c	‐			
	Autumn	0.80	0.16	0.13	a–c	‐			
	Winter	0.95	0.20	0.10	b–d	‐			

*Notes.* Note that the winter season of the Poverty Creeck was divided into two subperiods (winter 1 and winter 2) with contrasting characteristics, as detailed in the Supporting Information S1. The reported *R* ^2^ refers to the regression used for the estimation of parameters *a* and *b*.

### 4.1 Flow Duration Curves and Stream Length Duration Curves

**Figure 4:**
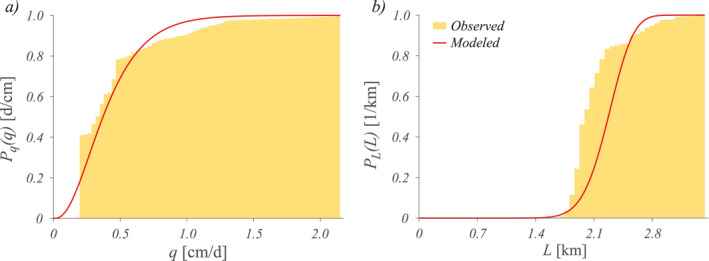
Cumulative distribution functions of streamflow (*P*_*q*_(*q*), panel (a)) and active length (*P*_*L*_(*L*), panel (b)) for the Valfredda site: comparison between models and observations.

In the Valfredda catchment, the effective rainfall frequency was moderate (one flow pulse every 7 d on average), whereas the observed recession rate was much lower, leading to an average response time of 20 d (Table 2). Despite the process complexity, the analytical model was able to capture reasonably well the streamflow statistics observed in the Valfredda. The analytical *P*_*q*_(*q*) underestimated low discharges and overestimated high streamflows, though reproducing in a reliable manner the shape of the observed cdf (Figure 4a). The MAE, evaluated as the mean difference between the modeled and the observed discharges associated to the 0.2, 0.4, 0.6, and 0.8 quantiles of the distribution, is 0.054 cm/d. The relatively low value of the SMAE (12.7%) indicates a good performance of the model in this case.

The shape of the discharge cdf mirrored the relatively high frequency of flow‐producing events and the importance of groundwater contributions to the discharge, which reduced the observed discharge variability and led to a persistent flow regime. This behavior complied with the climate of the region. In fact, most of the Alpine rivers in this area are characterized by a constant water supply during the summer and the early fall, which is due to the high frequency of rainfall events (Botter, Basso, Porporato, Rodriguez‐Iturbe, & Rinaldo, 2010).

The network extension scaling exponent *b*, which represents the river dynamicity, is equal to 0.17 indicating a reduced responsiveness of the active length to changes in the underlying climatic conditions. The relatively low value of the *R* ^2^ (*R* ^2^ = 0.52) is related to the scatter of the points around the regression line which is caused by the high variability of observed active lengths for a given discharge, especially during moderate rain events.

The analytical cdf of the active length (Equation 10) mirrors the behavior of the underlying streamflow distribution (Figure 4b). The plot shows that the modeled *P*_*L*_(*L*) underestimates low length probabilities and overestimates high length probabilities, but it is able to fit reasonably well the observed cdf of *L*. The MAE, evaluated as the difference between the analytical and the observed active length cdfs is relatively low and equal to 0.091 km. The performance of the analytical model was deemed satisfactory (SMAE = 14%), in the light of the simplicity of the approach, the limited number of the required parameters and the geological complexity of the study catchment. The shape of *P*_*L*_(*L*) suggested the presence of limited temporal changes in the active length, which are represented by a perennial active length regime. In fact, the presence of significant groundwater contributions to the discharge ensured a continuous supply to the perennial portion of the drainage network (1,045 m).

**Figure 5:**
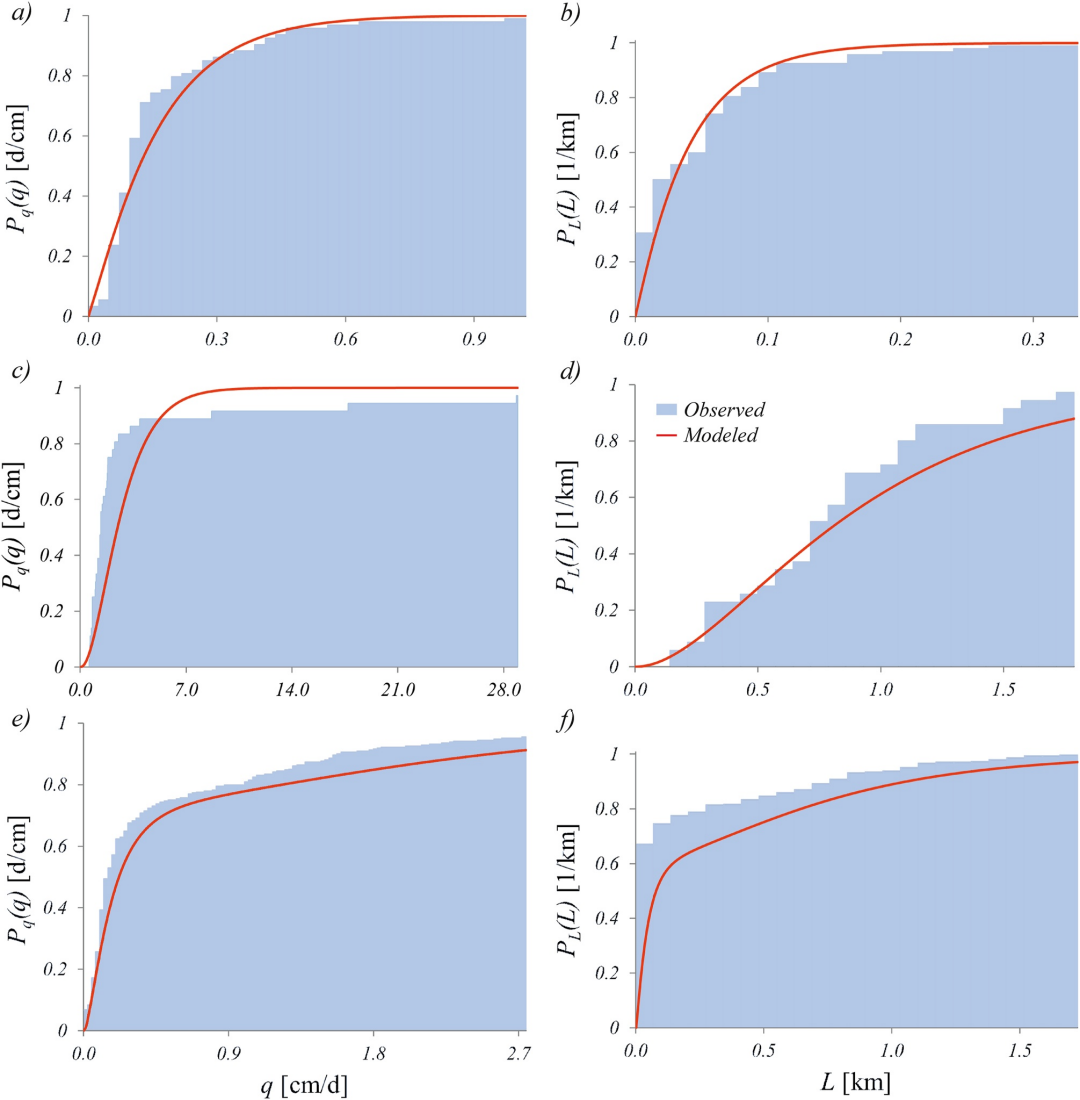
Cumulative distribution functions of streamflow (*P*_*q*_(*q*), left plots) and active length (*P*_*L*_(*L*), right plots) for the Poverty site. Panels (a and b) refer to the summer season, panels (c and d) to the spring season, while panels (e and f) refer to the whole year.

In the Poverty Creek, the mean recession rate varied between 0.2 and 0.12 d^−1^ depending on the time of the year, while the mean frequency of effective rain pulses was in the range (0.23–0.34 d^−1^). The mean intensity of effective rain events, instead, shows a marked variability across the seasons (Table 2). The annual streamflow cdf of the Poverty Creek has been calculated as a weighted average of the seasonal *P*_*q*_(*q*). The annual flow regime is influenced by the presence of seasonal droughts generally observed during the summer and the fall. During the summer, in particular, the ratio *λ*/*k* was slightly larger than 1, with modal discharge values close to zero and an intermediate‐to‐persistent flow regime (Figure 5a). Summer and autumn were characterized by high temperature and evapotranspiration rates, which induced low discharge rates and pronounced event‐based streamflow fluctuations. On the other hand during the spring the mean discharge was much higher, and the frequency of flow pulses was larger than the corresponding recession rate, leading to a persistent flow regime in which *P*_*q*_ was almost zero for *q* < 0.6 cm/d (Figure 5c). While the seasonal analytical distributions did not perfectly represent the observations across all the seasons, the annual *P*_*q*_(*q*; Figure 5e) was able to reproduce the overall shape of the observed cdf quite well, with a MAE of 0.1 cm/d in this case (12.8% of the mean discharge). Nevertheless, the model underestimated the probability of low streamflows. At the annual timescale, the streamflow regime of Poverty Creek had an erratic behavior with *CV*_*q*_ > 1 and high slopes of *P*_*q*_ especially for low values of *q*.

In Poverty Creek, the parameter *b* that modulates the active length vs. discharge relationship is equal to 1.09, leading to an almost linear relationship between these variables. Relatively high values of *b* imply a high dynamicity of the river network which is consistent with the observed seasonal variations of the flowing length in this case. In addition, the relatively high value of *R* ^2^ (0.77), indicates that the power law model provides a good representation of the observed *L* vs. *q* relationship.

Similarly to the annual *P*_*q*_, the analytical cdf of the active length at the annual timescale was calculated as a weighed average of the seasonal curves. The annual *P*_*L*_(*L*) underestimated the observed cumulative distribution of wet length, particularly for *L* < 1 km. The shape of the modeled cdf indicates that a high probability is associated to the almost complete network shrinking. Likewise, a wide range of active lengths was associated to the highest non‐exceedance probabilities. The MAE, which is equal to 0.138 km (38.5% of the mean), indicates discrepancies between the modeled and the observed active length cdfs. This performance was still deemed acceptable, as the overall shape of the cdf was captured by the model (Figure 5f). The shape of annual active length distribution was influenced by the seasonality of the climate in the study site. During the spring (Figure 5d) and the winter (Supporting Information S1), high values of the active stream length were observed, but no particular preferential states were observed in the network configuration. On the other hand, the summer (Figure 5b)—similarly to the autumn (Supporting Information S1)—was characterized by dry conditions with active lengths which were often close to zero (with *P*_*L*_ approaching 1 for *L* < 0.3 km).

**Figure 6:**
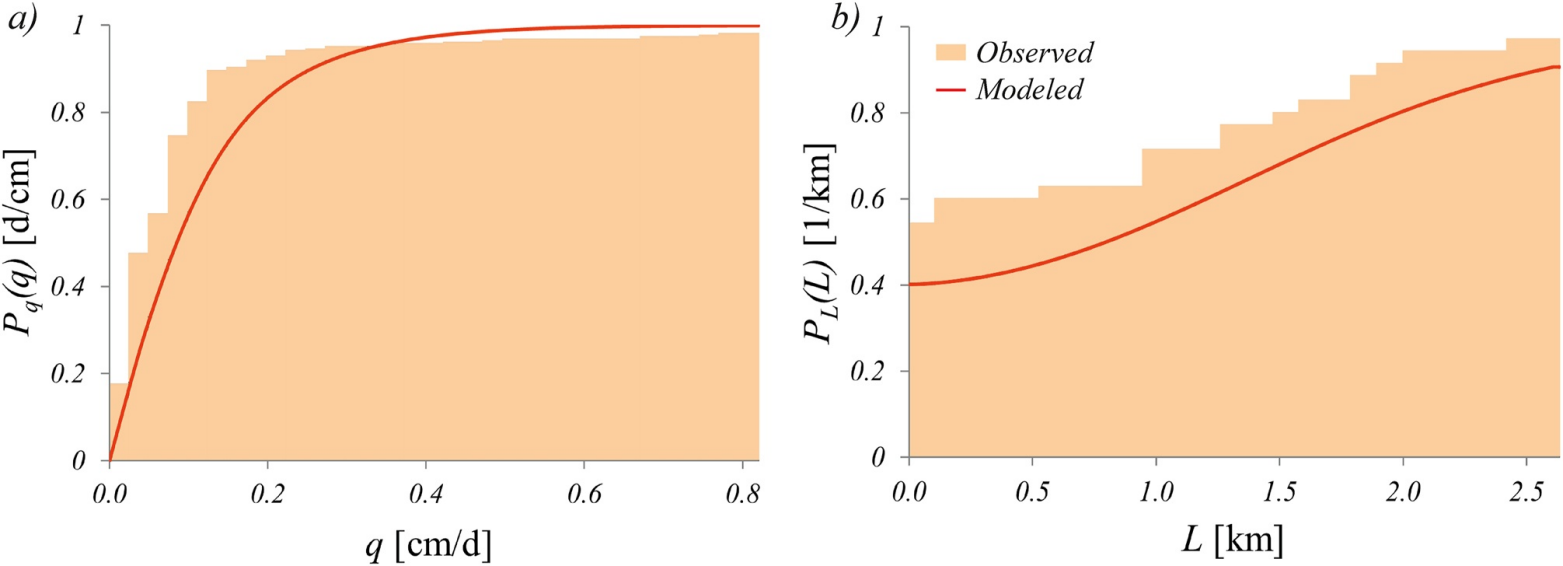
Cumulative distribution functions of streamflow (*P*_*q*_(*q*), panel (a)) and active length (*P*_*L*_(*L*), panel (b)) for the Turbolo site.

In the Turbolo catchment, owing to the strong seasonality in the underlying hydroclimatic conditions, the parameters *α*, *λ*, and *k* all varied significantly across the year. The mean effective rainfall depth was slightly lower than 1 cm/d in all seasons but the spring, during which *α* = 0.52 cm/d. The effective rainfall frequencies, instead, ranged from 0.04 d^−1^ during the summer to 0.20 d^−1^ during the winter and the spring. The mean recession rate followed a similar trend, with values that were in the range (0.06–0.13 d^−1^). The annual *P*_*q*_(*q*), calculated as a weighted average of the seasonal curves, slightly underestimated the probability of low streamflows but it was able to properly reproduce the shape of the observed cdf of *q* (Figure 6a). The MAE of the streamflow model is equal 0.030 cm/d (17.8% of the mean) in this case.

The annual cdf was a concave function of *q*. This circumstance is related to the high slope of the summer FDC in correspondence of *q* → 0. The annual cdf also mirrored the pronounced seasonality of the climate in the whole Crati region. Therein, the summer season was characterized by high temperature and high evapotranspiration rates, which led to a low mean discharge with pronounced streamflow variations. Instead, the other seasons of the year were featured by more frequent rainfall events which induced a streamflow regime characterized by more abundant water resources and reduced hydrologic fluctuations.

The scaling exponent *b* is equal to 0.50 in the Turbolo, a value which lies in the literature range. The low value of *R* ^2^ (*R* ^2^ = 0.21) is possibly due to the fact that discharge measurements were only available at the outlet of much larger catchment.

The annual modeled *P*_*L*_(*L*) is reported in Figure 6b, where a comparison with the corresponding observed distribution is shown. The modeled *P*_*L*_(*L*) generally underestimated the probabilities associated with all the observed active lengths but it properly replicated the shape of the observed cdf. The MAE, calculated as the mean difference between the modeled and the observed active lengths associated to the 0.2, 0.4, 0.6, and 0.8 quantiles of the distribution, is equal to 0.329 km (49.0% of the mean). The MAE for the Turbolo indicates a systematic underestimation of *P*_*L*_ throughout the *L* domain.

### 4.2 Streamflow and Active Length Regimes

**Figure 7:**
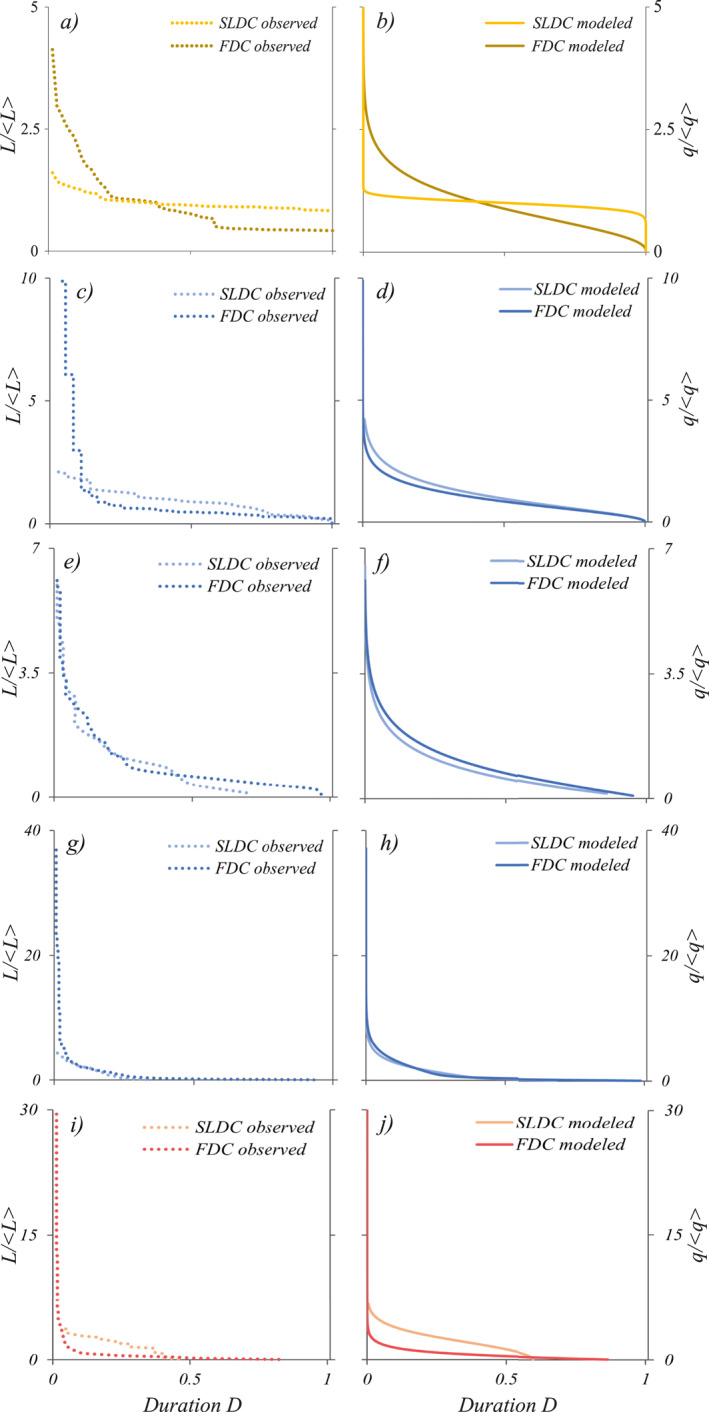
Flow duration curves (FDCs) and the stream length duration curve (SLDCs) using empirical data (left panels) and the corresponding analytical curves (right panels) for the three study catchments. Panels (a and b) refer to the whole study period in the Valfredda site. Panels (c–h) refer to different periods in the Poverty creek: spring (c, d), summer (e, f) and the whole year (g, h). Panels (i and j) show the annual curves for the Turbolo creek. All plots refer to dimensionless quantities (L* = *L*/〈*L*〉 and q* = *q*/〈*q*〉).

Figure 7 compares the shape of the FDC and the SLDC in the three study catchments, using both empirical data (left panels) and the corresponding analytical curves (right panels). The figure emphasizes that the analytical models are able to capture the observed relationship between the discharge and the active length regime across all the case studies.

The Valfredda river (Figures 7a and 7b) is characterized by a persistent flow regime and a perennial active length regime. In fact, the FDC displays a smooth shape and a relatively flat slope covering the full range of durations from 0 to 1. The SLDC has a smaller slope of the FDC, and displays a clear inflection point which makes the SLDC strictly positive for all the durations between 0 and 1. In this case, the ratio *λ*/*k* (= 2.8) is larger than 1 while *b* < 1. This implies that the Valfredda catchment, based on the theoretical regime classification proposed in Section 2.4, has a “class A” behavior. Accordingly, the theoretical and observed coefficients of variation of streamflows and wet lengths, turned out to be lower than 1, with *CV*_*L*_ < *CV*_*q*_ < 1. This behavior is mainly induced by the presence of few permanent groundwater springs feeding some perennial channels of the stream network.

Regarding Poverty Creek, the FDC and the SLDC both display pronounced seasonality (Supporting Information S1). In the spring (Figure 7c), the ratio *λ*/*k* is equal to 2.29, thereby suggesting a persistent flow regime, as mirrored by the knee‐shaped FDC. As the parameter *b* is equal to 1.09, the ratio *λ*/(*kb*) is also larger than 1. Therefore, the active length regime can be classified as a perennial regime, as reflected by the shape of the SLDC, which leads to positive values of *L*/〈*L*〉 for *D* → 1. Overall, the behavior during the spring belongs to the “class G”. However, during the summer, *λ*/*k* = 1.21 and *λ*/(*k b*) = 1.11. Under these circumstances (*λ*/*k* ≃ 1, *b* ≃ 1, *λ*/*k* > *b* and *λ*/*k* < 0.4114 *b* ^2^ + 0.7168 *b*), an ephemeral de facto active length regime and an intermediate flow regime are observed. Thus, during the summer, the behavior of Poverty Creek belongs to the “class F”.

The normalized annual FDC (Figures 7g and 7h) is quite steep and concave within the whole domain (0, 1). Similarly, also the annual SLDC has a steep shape and it clearly approaches the *x*‐axis at durations lower than 1, indicating an ephemeral active length regime. On an annual basis *CV*_*q*_ and *CV*_*L*_ are both larger than 1, indicating a pronounced variability of *q* and *L* in time, with a behavior in between “class C” and “class D” (*b* ≈ 1). This is mostly related to the high seasonality of discharge and active length regimes. In the Poverty Creek, the shape of the seasonal SLDCs resembles that of the corresponding FDCs, as *b* ≃ 1.

In the Turbolo, the streamflow variability is enhanced (*CV*_*q*_ > 1) and an erratic flow regime is observed, which is reflected by the convex shape of the FDC (Figures 7i and 7j). Similarly, also the temporal variations of the active length are pronounced (*CV*_*L*_ > 1). The annual SLDC intersects the *x*‐axis in a point with a duration lower than 1, suggesting an ephemeral active length regime. Overall, the Turbolo shows a “class C” behavior. The ephemeral nature of the active length regime in the Turbolo catchment is not surprising in the light of the climate of the study site. In several cases, the river network has been detected as completely dry, with active length values equal to 0. This is in accordance with the Mediterranean climate, which is characterized by high temperature and prolonged droughts during the whole summer, which leads to a seasonal dry‐down of the drainage network.

## 5 Discussion

The concept of SLDC was first introduced in Botter and Durighetto (2020) to efficiently summarize the dynamics of the active length. In Botter and Durighetto (2020), the relationship between the SLDC and local features of the stream network, such as the local persistency of surface flow and the spatial correlation of the nodes state (dry/wet) were examined. In this article, instead, we provide a novel functional form of the SLDC using a parsimonious approach and the concept of derived distribution, which can be used for practical applications to interpret key statistics of the active stream length. The modeling approach presented in this article can be applied in cases where the necessary hydroclimatic and flowing length data are available. The minimum data requirements for the adoption of the flow regime characterization used in the manuscript (i.e., the analytical expression of the FDC) is quite limited. While model performances improved when streamflow and rainfall records were combined in the parameter estimation procedure (as in this specific application), *λ*, *α*, and *k* could be also estimated in the absence of discharge timeseries from widely available climatic and landscape characteristics (rainfall, climate, a digital terrain map of the area, soil and vegetation features, see Doulatyari et al., 2015). On the other hand, the exponent of the power‐law model that drives the shape of the SLDC, *b*, requires a set of joint measurements of *L* and *q*. While the estimation of *L* from field surveys is highly time‐consuming, the ensuing data are considered to be quite robust, as most of the uncertainty relies on the practical definition of an “active stream” through the identification of threshold widths and/or velocities. In the literature, the parameter *b* has been often assessed using relatively few river network surveys (Godsey & Kirchner, 2014; Jensen et al., 2017) and its estimate seems to be relatively stable when the sampling frequency changes (Zanetti et al., 2021). Thus, we suggest that relatively few surveys (e.g., from 5 to 10) performed under different hydrologic conditions should be enough to estimate the value of the scaling exponent *b* and enable the extension of the analytical streamflow regime classification to the active length regime. In spite of the associated practical issues of their field deployment, the use of water presence sensors for monitoring changes in the flowing network length may be better suited to obtain unbiased sample statistics of *L*, because a continuous‐time monitoring of the configuration of the active network is allowed. This is particularly true for the highest values of *L*, which are observed only during the most intense rainfall events and are thus usually underrepresented by visual field surveys for practical reasons.

The simplicity of the proposed approach represents a prominent feature of the model, which makes it suited to describe analytically the statistics of the discharge and the active length of catchments that are forced by a stochastic sequence of rain events. In particular, the limited number of parameters involved—from 3, when the dimensionless discharge q* and active length L* are concerned, up to 5 or 6 for the corresponding dimensional quantities *q* and *L*—enables a parsimonious representation of the interactions among different types of hydroclimatic processes that underlie the observed network dynamics (e.g., rainfall, evapotranspiration, channel transmissivity, seepage). This also suggests the potential applicability of the model for the prediction of the changes in active length regimes produced by temporal shifts in landscape and climate characteristics.

The performance of the streamflow model was in line with previous applications (Botter et al., 2013), with an average SMAE (scaled mean absolute among 4 reference quantiles of the cdf of *q*) equal to 0.14. The main shortcomings of the streamflow model have been already discussed in the existing literature (Basso et al., 2015; Muller & Thompson, 2015) and mainly pertains to the lack of ergodicity of the timeseries, the presence of non‐linearity in the hydrological response and the non poissonianity of rainfall. In this study, model errors were larger in cases where the flow regime was erratic. If the flow regime was persistent, the presence of outliers in the available streamflow sample could deteriorate model performance (e.g., for the spring season of the Poverty Creek and for autumn season of the Turbolo).

Overall, the model performances for the active length module were lower than those of the discharge model, with an average SMAE across the case studies of 0.3. This was related to the fact that the derived distribution approach transfers all the inaccuracies of the streamflow model to the active length statistics. Furthermore, the presence of hysteresis in the *L* − *q* relationship, which could not be captured by our analytical framework, represented an additional source of errors in the application of the analytical model for *P*_*L*_. In agreement with the behavior of the streamflow model, the analytical expressions for the active length statistics led to higher errors in the ephemeral de facto and ephemeral active length regimes (e.g., annual period, summer season, and autumn season of Poverty Creek and annual period of the Turbolo). Therefore, the model obtained with the derived distribution approach seems to be better suited to simulating the active stream length statistics in rivers characterized by a moderate‐to‐low temporal variability of the flowing length. Overall, the reasonable performances of the length module suggest the potential and the robustness of the proposed tool for the characterization of flowing length regime of rivers. Further analyses would be however necessary to formally characterize model performances and the related uncertainty under a broader range of settings and quantify the bias possibly introduced by the limited sample size and the specific dates of the field surveys. This goes outside the scope of this article, and is deferred to upcoming publications.

The proposed approach allowed for the identification of two dimensionless parameters, namely *λ*/*k* and *b*, which can be used to provide a consistent and quantitative classification of the underlying streamflow and active length regimes. In particular, we have identified 7 classes of behavior, which correspond to different combination of flow regimes (erratic and persistent) and active length regimes (ephemeral, epral de facto, perennial). In line with the theory, the experimental case studies investigated in this article display a quite heterogeneous set of regimes, including most of the classes foreseen by the analysis presented in Figure 7 (namely, classes “A”, “C”, “D”, “F” and “G”). Nevertheless, further applications of this modeling approach to other sites with different hydroclimatic and landscape characteristics would be an important step forward to test the robustness of the proposed analytical method, and to verify the existence of all the different types of active length and streamflow regimes predicted by the classification developed in this article. This is the object of ongoing work.

## 6 Conclusion

In this article an analytical model for the characterization of the streamflow regimes was integrated with an empirical power law model that links active length (*L*) and specific discharge (*q*) to provide a probabilistic description of the temporal variations of the flowing length of a dynamic stream network. Novel analytical expressions have been derived for the probability density function of the flowing length and for the SLDC based on five hydroclimatic and morphological parameters. These expressions link the statistics of the active stream length to a set of relevant hydrological processes including rainfall dynamics, recession characteristics, and the sensitivity of the active length to changes in the catchment discharge.

The model has been tested using data from three different headwater catchments located in Italy and in the USA. The selected case studies provide representative scenarios characterized by different hydrologic conditions (e.g., temperature, rainfall, evapotranspiration, vegetation, and soil/geologic properties). Model performances were satisfactory with a mean of the annual SMAEs across the case studies of 15% for the FDC and 33.0% for the SLDC. Larger errors were typically associated to drier climates and highly dynamic stream networks.

The theoretical analysis proposed in the article revealed that streamflow and active length regimes are only dependent on two dimensionless parameters: (a) the ratio *λ*/*k* between the frequency of flow pulses and the catchment recession rate; (b) and the scaling exponent *b* that regulates the relationship between *L* and *q*. In particular, analytical arguments and empirical evidence indicate that, depending on the value of the dimensionless parameters *λ*/*k* and *b*, seven different classes of behavior can be identified (from “class A” to “class G”). These classes correspond to different combinations of streamflow regimes (erratic or persistent) and active length regimes (perennial, ephemeral de facto, ephemeral). In general, when *b* < 1 (*b* > 1), the SLDC is flatter (steeper) than the corresponding FDC. On the other hand, when *b* ≃ 1 the shape of the FDC and that of the SLDC are similar. The variety of hydrological behaviors predicted by the theory is also confirmed by the analysis of the empirical data collected in our selection of cases studies.

The analyses presented in this article suggest that the proposed model and the implied joint classification from the flow and active length regimes might be useful tools for an objective estimation of the joint dynamics of *q* and *L* in catchments characterized by diverse climate, geological and morphological features.

## 7 Symbol List

**Notation**
〈⋅〉: averaged quantity.
***L***
: active flowing length.
*q*
: specific discharge (per unit catchment area).
FDC: flow duration curve.
SLDC: stream length duration curve.
*λ*
: frequency of flow pulses.
*k*
: recession time constant.
*λ*_*p*_
: frequency of rainfall.
*α*
: average daily rainfall depth.
Γ: complete Gamma‐function.
*γ*
: lower incomplete Gamma‐function.
*CV*
: coefficient of variation (standard deviation scaled to the mean).
*a*
: proportionality constant of the relationship that links active length and catchment discharge.
*b*
: scaling exponent of the relationship that links active length and catchment discharge.
*q*_0_
: threshold specific discharge for which *L* = 0.
*D*
: duration (percentage of time).
*p*_*L*_(*L*): probability density function of *L*.
*p*_*q*_(*q*): probability density function of *q*.
*P*_*L*_(*L*): cumulative distribution (i.e., non‐exceedance probability) of *L*.
*P*_*q*_(*q*): cumulative distribution (i.e., non‐exceedance probability) of *q*.
*L*_0_
: modal value of *L*.
$p_{L}^{c}$
: continuous part of the pdf of *L*.
*δ*
: Dirac delta function.
q*: normalized discharge (ratio between discharge values *q* and mean discharge 〈*q*〉)
L*: normalized active length (ratio between active length values *L* and mean active length 〈*L*〉)
MAE: mean absolute error.
SMAE: scaled mean absolute error (MAE scaled to the mean).
*R* ^2^
: coefficient of determination.

## Erratum

In the originally published version of this article, we failed to accurately acknowledge that a previous paper derived an equation very similar to our Equation (15). The text just prior to Equation (15) now reads, “Similarly, the coefficient of variation of the active length can be analytically derived as (Lapides et al., 2021):”, and this version may be considered the authoritative version of record.

## Supporting information

Supporting Information S1

## Data Availability

All the empirical data used in this study is publicly available, see citations in text.

## Appendix A The Stream Length Duration Curve (SLDC) With Permanent Streamflow Component

When the active network length is equal to 0 even if the corresponding observed discharge is positive (see Equation 7), Equation 7 reads:

(A1)
$$q={\left(\frac{L}{a}\right)}^{\frac{1}{b}}+q_{0}.$$

Therefore, the cumulative distribution function of *L* for this specific case can be written as:

(A2)
$$P_{L}\left(L\right)=\frac{\gamma \left(\frac{\lambda }{k},\;\left({\left(\frac{L}{a}\right)}^{\frac{1}{b}}+q_{0}\right)\frac{1}{\alpha k}\right)}{\Gamma \left(\frac{\lambda }{k}\right)}.$$

In line with the previous derivations, the pdf of *L* when *q*_0_ ≠ 0 is obtained by differentiating Equation A2 as:

(A3)
$${p_{L}}^{C}\left(L\right)=\frac{{\left(\alpha k\right)}^{-\frac{\lambda }{k}}}{ab\Gamma \left(\frac{\lambda }{k}\right)}{\left(\frac{L}{a}\right)}^{\frac{1-b}{b}}{\left({\left(\frac{L}{a}\right)}^{\frac{1}{b}}+q_{0}\right)}^{\frac{\lambda }{k}-1}\exp\left[-\left({\left(\frac{L}{a}\right)}^{\frac{1}{b}}+q_{0}\right)\cdot \frac{1}{\alpha k}\right].$$

Equation A3 is valid only for *L* > 0 and represents the continuous part of the pdf, $p_{L}^{C}$. However, in this case, a finite probability is associated to *L* = 0. This corresponding “atom” of probability in *L* = 0 must be added to the pdf so as to account for the fact that the network is completely dry when *q* ≤ *q*_0_. The atom of probability can be written as:

(A4)
$${p_{L}}^{A}\left(L\right)=P_{q}\left(q_{0}\right)\delta \left(L\right),$$

where $P_{q}\left(q_{0}\right)$ = Prob[*q* ≤ *q*_0_] as given by Equation 3 and *δ* is the Dirac delta function. The overall pdf of the active length, in this case, can be expressed as a sum between the continuous part of the pdf and the atom:

(A5)
$$p_{L}\left(L\right)={p_{L}}^{C}\left(L\right)+{p_{L}}^{A}\left(L\right).$$

The SLDC can be obtained by inverting the function $P_{L}\left(L\right)$ as given by Equation 10 and Equation A2.
