# Supplementary material for: Probabilistic Description of Streamflow and Active Length Regimes in Rivers
Source: Water Resour Res. 2022 Apr 8;58(4):e2021WR031344. doi: 10.1029/2021WR031344 (PMC9286364; doi:10.1029/2021WR031344)
Supplement: Supplementary file 1 — Supporting Information S1 [file WRCR-58-0-s001.pdf]

Supporting Information for "Joint probabilistic description of streamflow and active length regimes in temporary streams"

Nicola Durighetto[1], Veronica Mariotto[1], Francesca Zanetti[1], Kevin J. McGuire[2], Alfonso Senatore[3], Gianluca Botter[1]

[1]Department of Civil, Environmental and Architectural Engineering, University of Padua, Italy.

[2]Dept. of Forest Resources & Environmental Conservation, Virginia Tech, VA, USA.

[3]Department of Environmental Engineering, University of Calabria, Italy.

S1. Seasons' delineation

Active stream length and discharge data for the Valfredda catchment refer to the late summer and the fall season, between september 4 and october 24, 2019.

The available data for the Poverty Creek catchment were taken between April 2017 and February 2018. The dataset was then subdivided into four different seasons: i) spring (from the 27th of April 2017, when the data collection started, to the 31st of May); ii) summer (from the 1st of June to the 31st of August 2017); iii) autumn (from the 1st of September to the 30th of November 2017); iv) winter (from the 1st of December to the 27th of February 2018). the observed time series of rainfall and streamflows suggest that the winter season has a bimodal behaviour: from the 1st of December to the 11th of January, the catchment shows a predominantly dry hydrologic regime, while from January 12th till February 27th 2018 the regime becomes wetter. For this reason, the statistics of q and L have been studied separately for the the above mentioned winter subperiods.

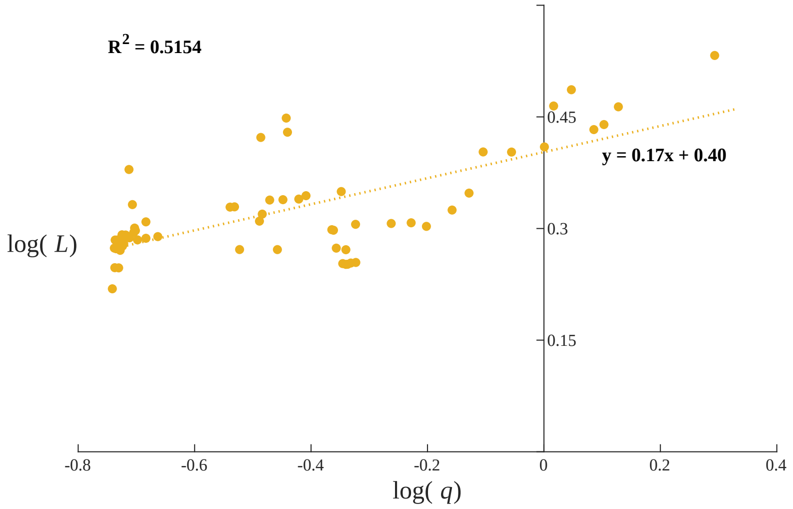

Figure S1. Valfredda log(L) vs log(q) plot including the regression line with its equation and the R^2.

For the Turbolo catchment data were collected from April 2019 to January 2020, and four different seasons were identified. Spring goes from the 1st of April 2019 to the 31st of May 2019, the summer season refers to the period from the 1st of June 2019 to the 31st of August 2019 while autumn goes from 1st of September to the 30th of November 2019. Winter goes from the 1st of December 2019 to the 31st of January 2020.

S2. Parameters of the length - streamflow relationsphip

The logarithmic plots that show the pairs of (q, L) data for the three case studies are reported in Figure S1, Figure S2 and Figure S3).

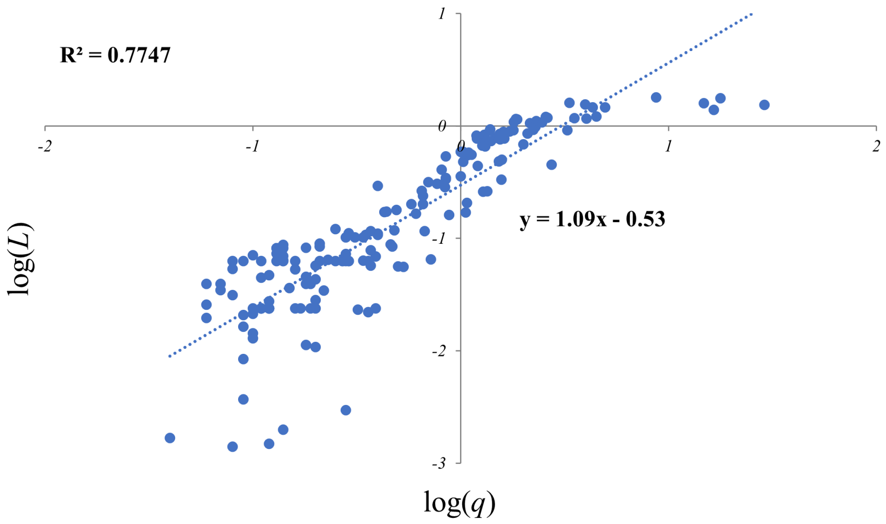

Figure S2. Poverty Creek $\log(L)$ vs $\log(q)$ plot including the regression line with its equation and the R^2.

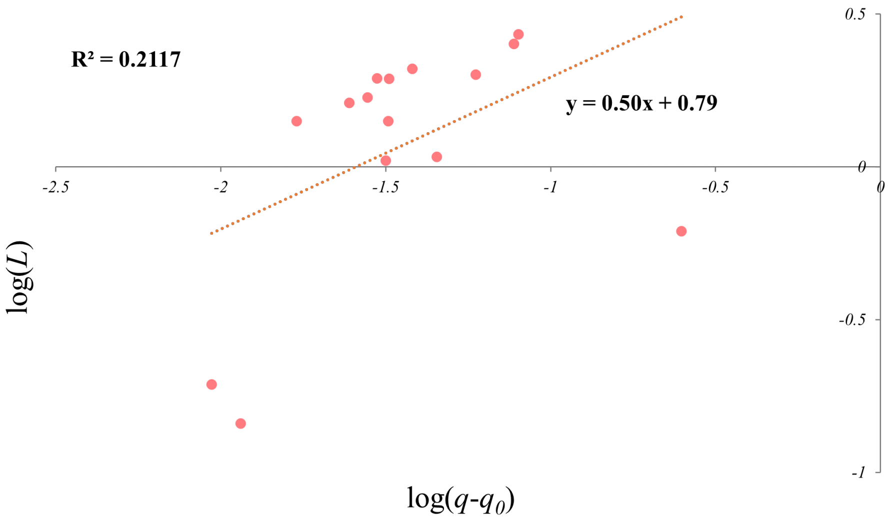

Figure S3. Turbolo $\log(L)$ vs $\log(q - q_0)$ plot including the regression line with its equation and the R^2.

S3. Autumn and winter cdfs of the Poverty Creek catchment

Observed and modeled cumulative density functions of active length and discharge for the autumn and winter seasons in the Poverty Creek catchment are shown in Figures S4 and S5).

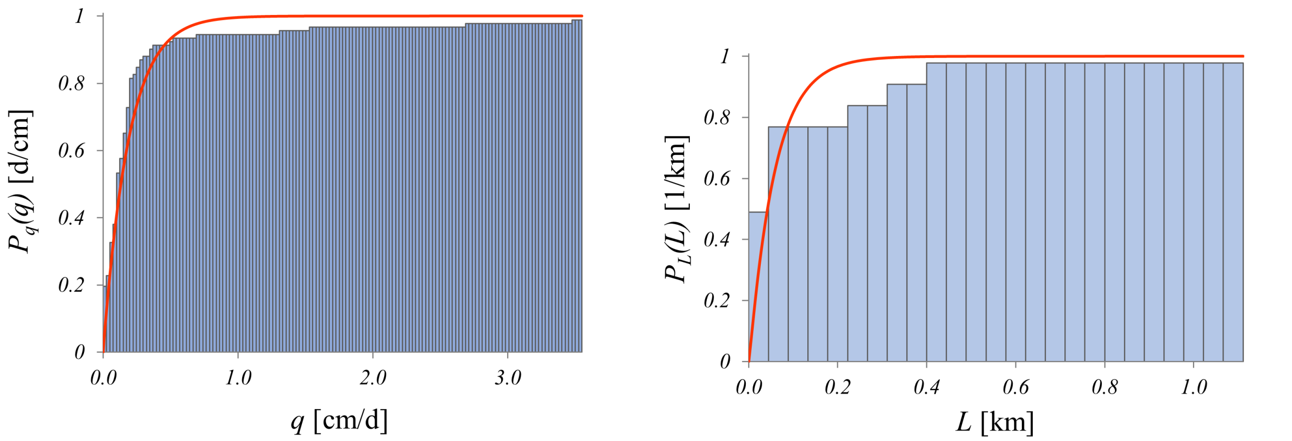

Figure S4. Poverty Creek observed (bars) and modeled (solid line) $P_q(Q)$ (left) and $P_L(L)$ referred to the autumn season.

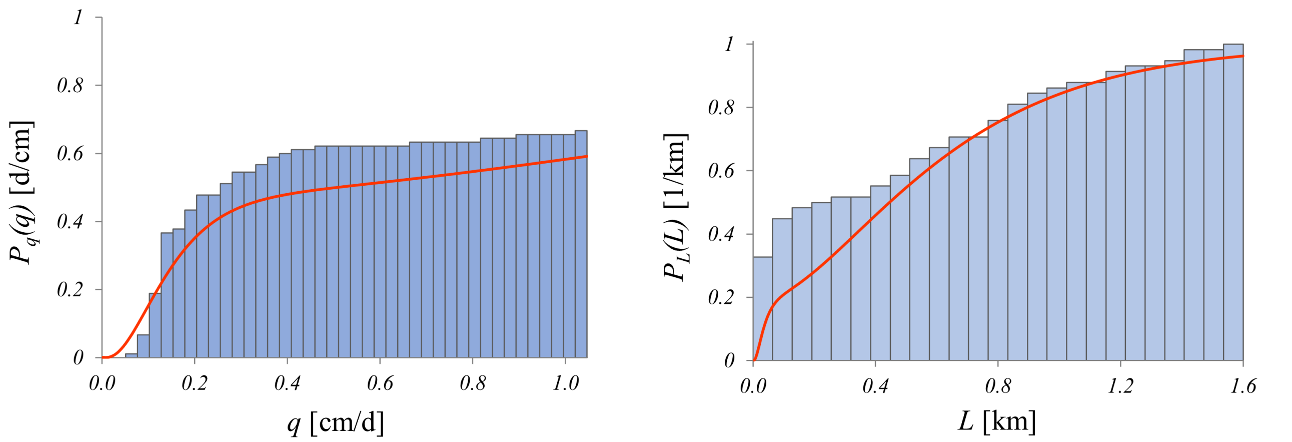

Figure S5. Poverty Creek observed (bars) and modeled (solid line) $P_q(Q)$ (left) and $P_L(L)$ referred to the winter season.

S4. Autumn and winter SLDC and FDCs of the Poverty Creek catchment

Modeled and observed duration curves of streamflows and active lengths of the two seasons not reported in the main text for the Poverty Creek catchment are reported in Figures S6 and S7).

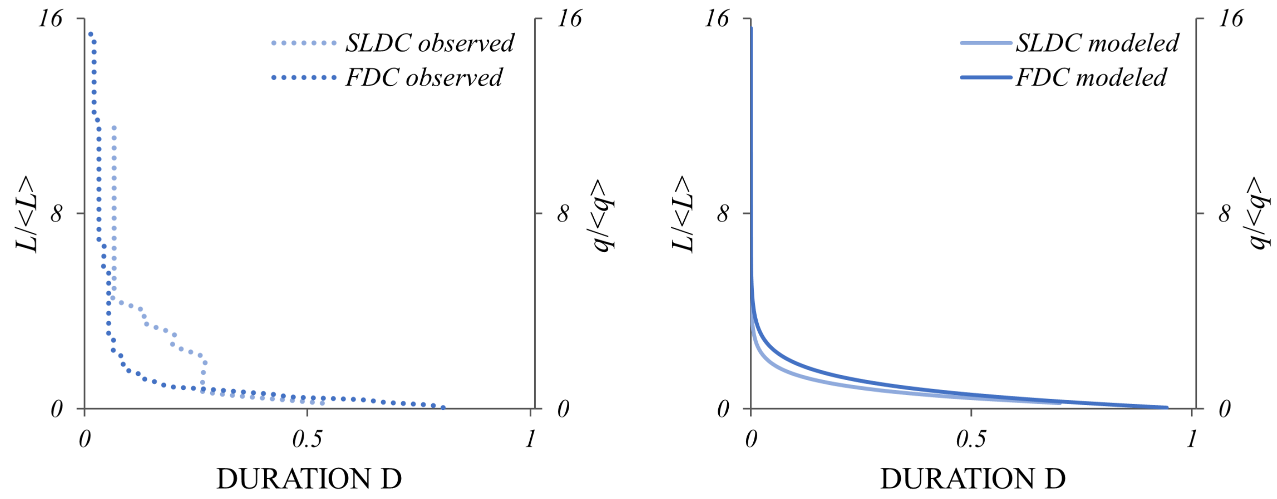

Figure S6. Poverty Creek observed (dotted line) and modeled (solid line) normalized FDC and SLDC referred to the autumn season.

S5. Probability density functions of q and L for the three case studies

Observed and modeled probability density functions of the three case studies for all the seasons are represented in Figures S8, S9, S10, S11, S12 and S13).

Note that the pdf of the active lengths for the Turbolo catchment has been assessed only at the annual time scale, owing to the limited amount of available L data during individual seasons. Moreover, as several wet length data were equal to 0 even when the observed streamflow was larger than 0, the analytical $p_L(L)$ was obtained through Eq.(22) in the main text. Hence the

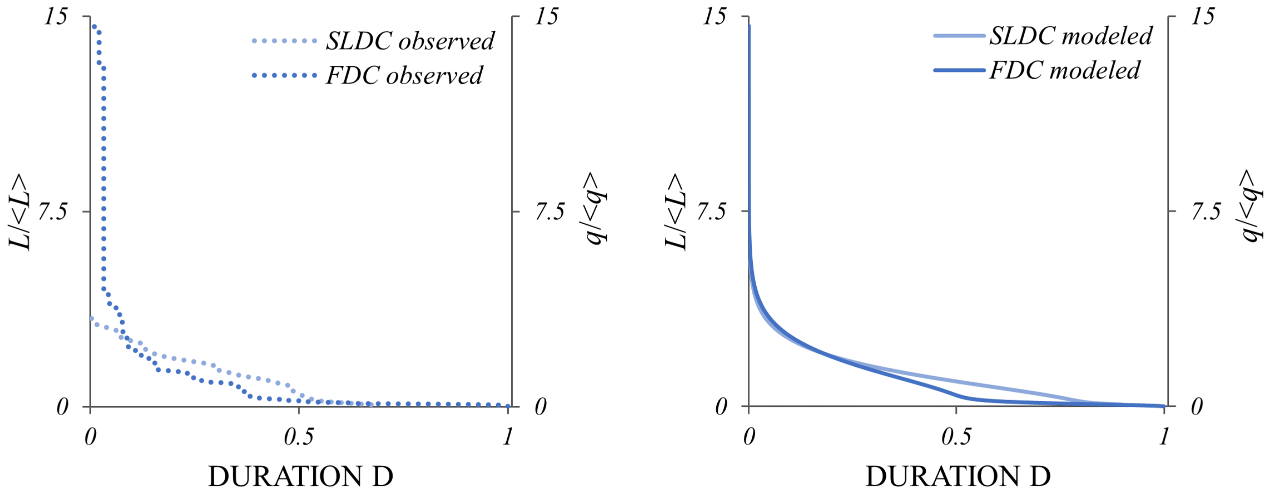

Figure S7. Poverty Creek observed (dotted line) and modeled (solid line) normalized FDC and SLDC referred to the winter season.

pdf is made of parts; the continuous part, indicated with $p_L^C(L)$ and the one that represents the atom of probability for $L = 0$, indicated with $p_L^A(L)$, both represented in Figure S13.

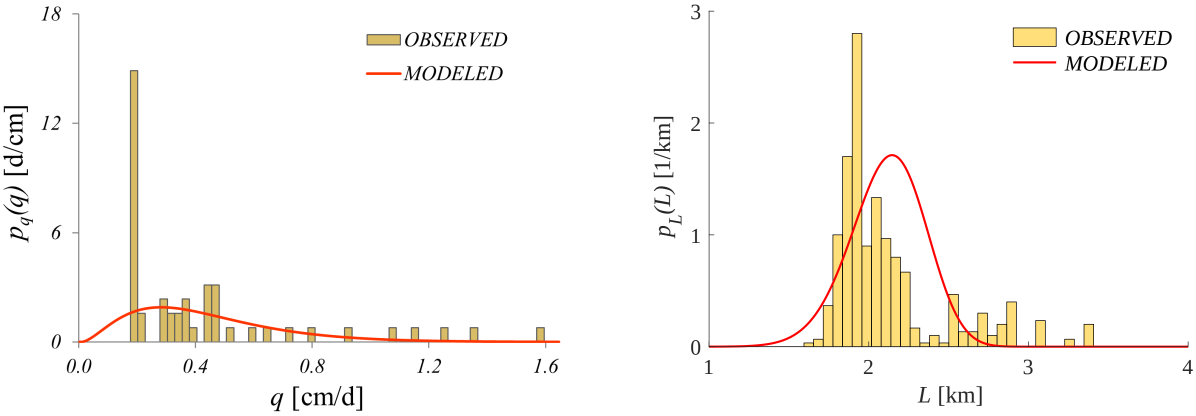

Figure S8. Valfredda observed (bars) and modeled (solid line) $p_q(q)$ (left) and $p_L(L)$ (right) for the overall period.

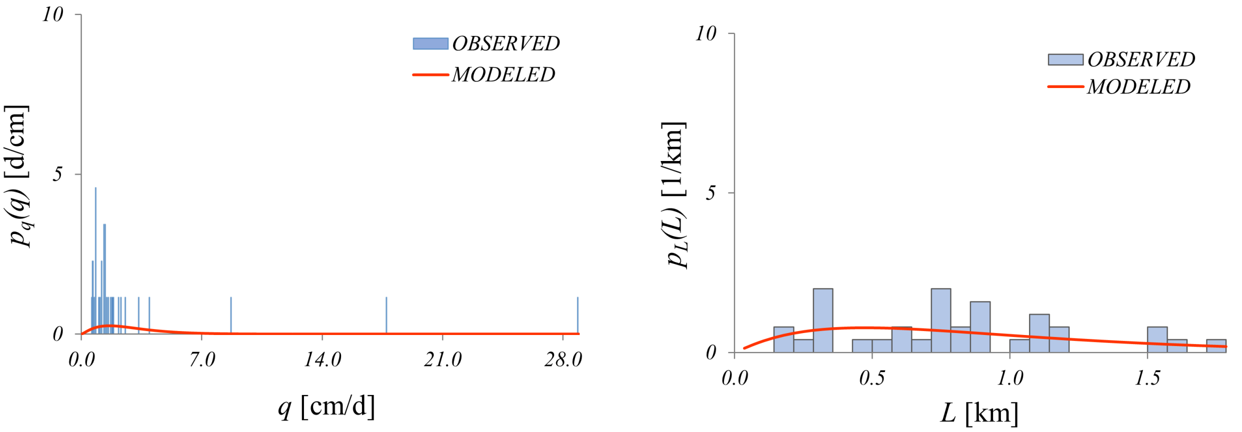

Figure S9. Poverty Creek observed (bars) and modeled (solid line) $p_q(q)$ (left) and $p_L(L)$ (right) for the spring season.

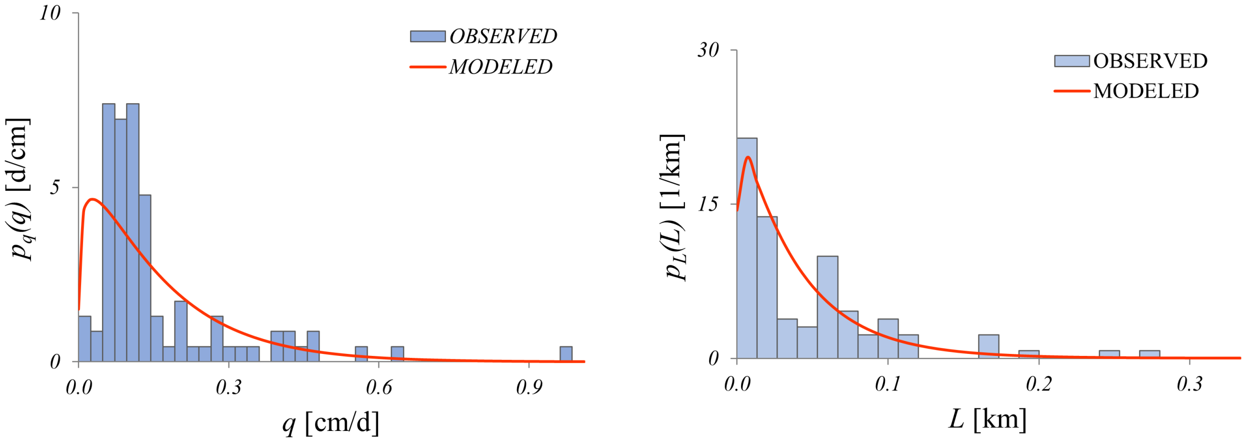

Figure S10. Poverty Creek observed (bars) and modeled (solid line) $p_q(q)$ (left) and $p_L(L)$ (right) for the summer season.

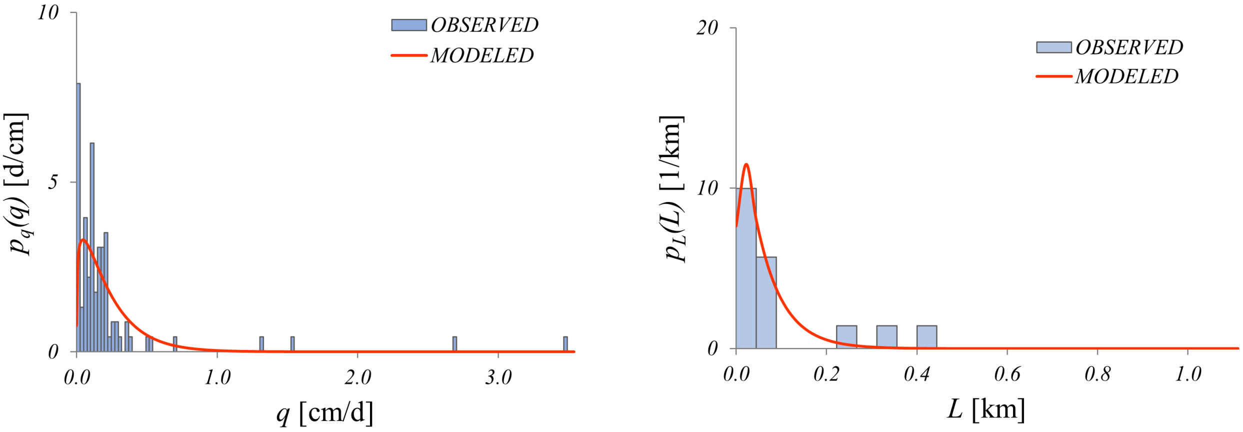

Figure S11. Poverty Creek observed (bars) and modeled (solid line) $p_q(q)$ (left) and $p_L(L)$ (right) for the autumn season.

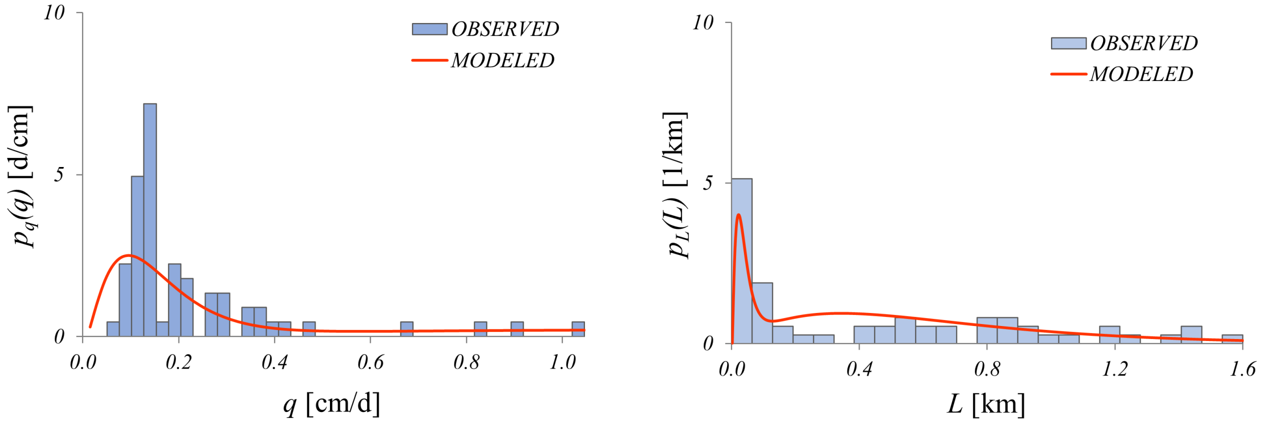

Figure S12. Poverty Creek observed (bars) and modeled (solid line) $p_q(q)$ (left) and $p_L(L)$ (right) for the winter season.

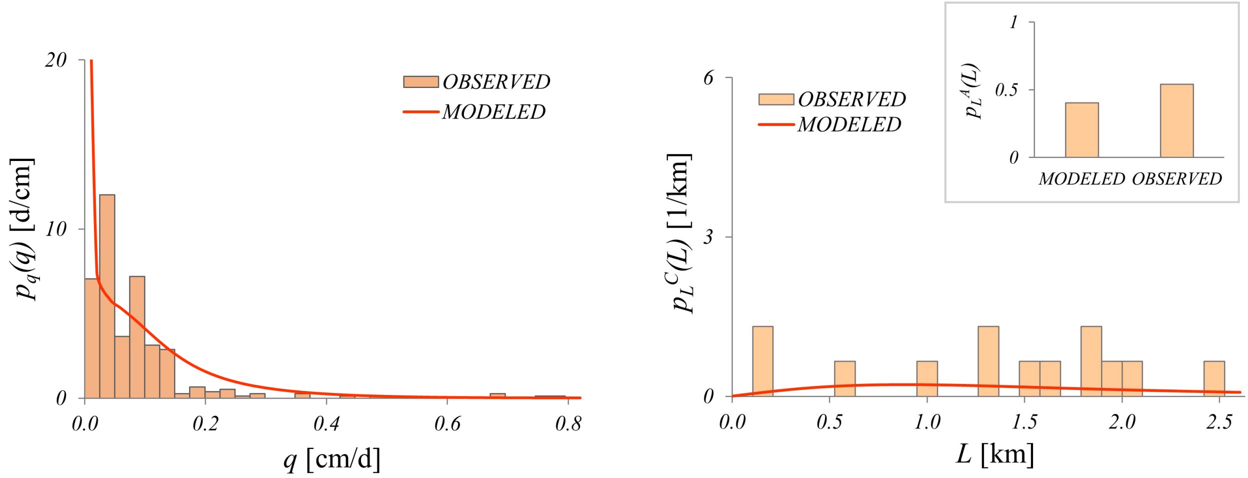

Figure S13. Turbolo observed (bars) and modeled (solid line) $p_q(q)$ (left), $p_L^C(L)$ and $p_L^A(L)$ (right) for the overall period.
